# Composites containing resins and carbon nano-onions as efficient porous carbon materials for supercapacitors

**DOI:** 10.1038/s41598-023-33874-w

**Published:** 2023-04-24

**Authors:** Gabriela Siemiaszko, Joanna Breczko, Agnieszka Hryniewicka, Anna Ilnicka, Karolina H. Markiewicz, Artur P. Terzyk, Marta E. Plonska-Brzezinska

**Affiliations:** 1 Department of Organic Chemistry, Faculty of Pharmacy with the Division of Laboratory Medicine, Medical University of Bialystok, Mickiewicza 2A, 15-222 Bialystok, Poland; 2 Faculty of Chemistry, University of Bialystok, Ciolkowskiego 1K, 15-245 Bialystok, Poland; 3 Faculty of Chemistry, Nicolaus Copernicus University in Torun, Gagarin 7, 87-100 Torun, Poland

**Keywords:** Chemistry, Energy science and technology

## Abstract

Herein, we report the functionalization of carbon nano-onions (CNOs) with the hydroxyaryl group and subsequent modifications with resins: resorcinol–formaldehyde using porogenic Pluronic F-127, resorcinol–formaldehyde-melamine, benzoxazine made of bisphenol A and triethylenetetramine, and calix[4]resorcinarene-derived using F-127. Following the direct carbonization, extensive physicochemical analysis was carried out, including Fourier transform infrared, Raman and X-ray photoelectron spectroscopy, scanning and transmission electron microscopy, and adsorption–desorption of N_2_. The addition of CNO to the materials significantly increases the total pore volume (up to 0.932 cm^3^ g^−1^ for carbonized resorcinol–formaldehyde resin and CNO (RF-CNO-C) and 1.242 cm^3^ g^−1^ for carbonized resorcinol–formaldehyde-melamine resin and CNO (RFM-CNO-C)), with mesopores dominating. However, the synthesized materials have poorly ordered domains with some structural disturbance; the RFM-CNO-C composite shows a more ordered structure with amorphous and semi-crystalline regions. Subsequently, cyclic voltammetry and galvanostatic charge–discharge method studied the electrochemical properties of all materials. The influence of resins' compositions, CNO content, and amount of N atoms in carbonaceous skeleton on the electrochemical performance was studied. In all cases, adding CNO to the material improves its electrochemical properties. The carbon material derived from CNO, resorcinol and melamine (RFM-CNO-C) showed the highest specific capacitance of 160 F g^−1^ at a current density of 2 A g^−1^, which is stable after 3000 cycles. The RFM-CNO-C electrode retains approximately 97% of its initial capacitive efficiency. The electrochemical performance of the RFM-CNO-C electrode results from the hierarchical porosity's stability and the presence of nitrogen atoms in the skeleton. This material is an optimal solution for supercapacitor devices.

## Introduction

Modern societies depend on fossil fuels and suffer from all problems related to pollution, global warming, increasing fuel costs, and geopolitical issues. Due to the increasing demand for high-power efficient energy storage, electrochemical supercapacitors (SCs) development has attracted much attention in recent years. The main reason is that SCs have many applications in industrial fields, mainly for the automotive industry (i.e., electric vehicles) and military purposes^1–3^. The SC devices can operate at high power rates compared to batteries^4–7^. However, the charge they can store is 3–30 times lower^5,7,8^. SCs are attractive because they offer unique solutions better than electrolytic capacitors and batteries, characterized by different storage mechanisms. Technical drawbacks of conventional storage devices are limited capacity and life storage. Therefore, many efforts were made to discover SC of high power density, low input resistance, extended lifetime, quick charge–discharge, and environmental friendliness^8–12^. The most promising materials seem to be carbon nanomaterials^9,13^, conducting polymers^14,15^, metal oxides^16,17^, and their composites^18^, and some less studied materials like covalent organic frameworks or metal–organic frameworks^19,20^, black phosphorus, or metal nitrides^21,22^.

Carbon materials are widely used in capacitors because of their morphological versatility and low cost^8,9,23^. In this group, carbon nanostructures (CN) exhibit many features like different shapes, sizes, hybridization states, heteroatom content, and microtexture, which play a crucial role in the properties and specific applications^24,25^. High surface areas, pores of sizes suitable to store different ions, and electrodes' polarizability and electrical conductivity are crucial for charging electric double layers (EDL) efficiently^4^. The most studied forms of nanocarbon being close to finding practical applications in electric capacitor devices are graphene^26,27^, carbon nanotubes (CNTs)^28–30^, and carbon nano-onions (CNOs)^31–33^.

CNOs are carbon allotropes possessing a unique structure with concentric graphitic layers^34^. Non-modified CNOs exhibit a specific surface area calculated by using the Brunauer–Emmett–Teller model (*S*_*BET*_) equal to 380–520 m^2^ g^−1^ depending on the annealing temperature and the specific capacitance (*C*_*S*_) value of 30 F g^−1^ ^31^. SCs are characterized by easy access for ions at the interface between carbon and electrolyte, high power density, and almost unlimited cyclability^35,36^. Many CNO-derived materials exhibit excellent electrochemical performance^31,37,38^. For example, chemical activation of carbon nanostructures can afford a few times larger capacity than non-modified ones^33^. The CNO surface, rich in unsaturated bonds, allows for their functionalization with various functional groups enhancing electrochemical properties.

It was reported by Velásquez et al. that the presence of pyrene moieties on the CNO surface caused the increase of the *C*_*S*_ by ca. 138%^39^. Interestingly, Zhang et al. described the preparation of a CNO/graphene hybrid as an ultrahigh-rate SC. The ultrafast ion transportation performance was possible due to the structural interconnection between the CNO, their surface curvature, and the covalent bonding between graphene and CNO^40^. Furthermore, the porous texture of the composites enhances their electrochemical performance and specific capacitance^41,42^. The nanostructural polyaniline, synthesized as nanotubes, used for the preparation of the composite containing conducting polymer and the oxidized CNO, allows obtaining material with more than three times higher capacitance than amorphous polyaniline reaching 946 F g^−1^ ^43^.

The preparation of porous carbon materials by pyrolysis of a polymeric network assembled on nanocarbon and its application as an efficient material for SC was performed. The synthesis of well-controlled porous carbon materials may be achieved by hard- or soft-templating processes^44^. The soft-templating method involves the application of the carbon-source component (*i.e.,* phenolic resin) in the presence of the pore-forming agent (*i.e.,* surfactants) or the application of some specific block copolymers^45,46^. The hard-templating is based on synthesizing the carbon-source component with the presynthesized hard templates (*i.e.*, silica), carbonization, and removal of the template^45–47^. Activation methods with oxidizing gases or chemicals are frequently used to enhance the porosity^47^.

Carbon nanostructures used as electrodes in SCs are mainly graphene^48,49^, CNTs^50,51^, and fullerenes^52^. The application of CNOs is very rarely reported. For example, Fulvio et al. described the synthesis and pyrolysis of nanocomposite made of phenolic resin, Pluronic F-127, and CNO and its further evaluation for energy storage^53^. They showed that the resistivity and capacitance of final carbon materials could be modified due to changing the ratios of resin to CN. However, the range of CNO content was high, equal to 5–75 wt%, in the starting synthesized gels before the thermal treatments. Furthermore, our group developed polymer-templated carbon materials derived from star-block copolymers and CNOs^54,55^. The hierarchical distribution of CN, in the amount of only ca. 5%, in a porous carbon matrix was responsible for increasing of the porosity and *C*_*S*_ value compared to pristine pyrolyzed polymers^54^.

Herein, we report the CNO functionalization with the hydroxyaryl groups and its involvement in the syntheses of resins organized on the surface of the nanostructural carbon: (1) resorcinol–formaldehyde with the application of porogenic Pluronic F-127, (2) resorcinol–formaldehyde-melamine, (3) benzoxazine made of bisphenol A and triethylenetetramine, and (4) calix[4]resorcinarene-derived resin with the application of F-127. Such an approach allowed for synthesizing materials with different porosity and amount of N atoms in the carbonaceous skeleton. Following the direct carbonization, all materials were characterized by several physicochemical methods and were examined as materials for SC. To our knowledge, the synthesis of CNO-doped porous carbons of benzoxazine type or derived from calix[4]resorcinarene or melamine has never been reported.

## Experimental section

### Materials

Commercially available nanodiamond (ND) powder with a crystal size between 4 and 6 nm (Carbodeon μDiamond®Molto and ND content greater than 97 wt%) was used for the preparation of the CNO nanostructures. The modified Kuznetsov method for the preparation of CNO by applying an annealing treatment under an inert atmosphere and reduced pressure of ultradispersed ND particles was used^37,56^. CNO were dried in a furnace at 120 °C overnight before use. DMF (POCH S.A., Poland) was distilled over P_2_O_5_ (pure, Honeywell, USA), and dried over molecular sieves 4 Å (POCH S.A., Poland) before use. Na_2_CO_3_ (≥ 99%, Aldrich, Germany), NaOH (97%, Aldrich, Germany), resorcinol (99%, Aldrich, Germany), HCHO (37 wt% in H_2_O, Aldrich, Germany), Pluronic F-127 (PEO_106_-PPO_70_-PEO_106_, Mw = 12,600 g mol^−1^, pure, Aldrich, Germany), melamine (99%, Aldrich, Germany), bisphenol A (97%, Aldrich, Germany), triethylenetetramine (TETA, mix of isomers, Aldrich, Germany), *p*-aminophenol (≥ 98%, Aldrich, Germany), NaNO_2_ (pure, Biomus, Poland), NaN_3_ (pure, Aldrich, Germany), benzaldehyde (≥ 99%, Aldrich, Germany), silica gel (0.040–0.063 mm, Merck, Germany), hexanes (Stanlab, Poland), ethyl acetate (AcOEt, POCH S.A., Poland), HCl (35–38%, Chempur, Poland), toluene (Stanlab, Poland), MeOH (Chempur, Poland), EtOH (POCH S.A., Poland), dioxane (Stanlab, Poland) were used as received. Water was distilled using DE 10 Plus distiller. *p*-Azidophenol, phenylcalix[4]resorcinarene, resorcinol–formaldehyde resin (**RF**), resorcinol–formaldehyde-melamine resin (**RFM**), benzoxazine resin (**BX**), and the phenylcalix[4]resorcinarene-derived resin (**CLX**) were synthesized using adapted literature procedures (detailed procedures are given in SI). Deuterated solvents, chloroform-*d* (CDCl_3_), and dimethylsulfoxide-*d*_*6*_ (DMSO-*d*_*6*_) were purchased from Euroisotop (United Kingdom).

### Methods

High-resolution transmission electron microscopy (HRTEM) was performed using a Titan G2 HRTEM microscope (FEI Company) equipped with a field emission gun (FEG). The electron beam accelerating voltage was 300 kV. HRTEM imaging of the sample microstructure was performed in a bright field mode using a CCD camera as detector. Before analysis, the samples were ground in an agate mortar to a fine powder. Into the obtained powder, 99.8% EtOH (POCH, Poland) was poured to form a dispersion, which was placed in an ultrasonic homogenizer for 10 s. The resulting slurry was taken with a pipette and placed on the Cu grids (200 mesh/inch) coated with carbon-stabilized formvar (Ted Pella, USA) until the solvent was evaporated.

The scanning electron microscope (SEM) measurements were performed using an INSPECT S50 microscope (FEI, Japan). The accelerating voltage of the electron beam was 15 keV. Before the measurements, an Au layer with a thickness of 7 nm was sputtered on the surface of the analyzed materials that formed a film on the conductive carbon base.

X-ray photoelectron spectroscopy (XPS) was performed using an ultrahigh vacuum chamber (PREVAC) with base pressure below 10^−8^ mbar using an Al Kα nonmonochromatic radiation source (1486.7 eV; 12 kV; 12 mA; VG Scienta SAX 100) and monochromator (VG Scienta XM 780). Detection of emitted photoelectrons was performed using a Scienta R4000 hemispherical analyzer. A low-resolution survey run (0–1200 eV) at pass energy of 200 eV was carried out. The C1s, O1s, and N1s high-resolution spectra were recorded at pass energy of 50 eV at RT. All the spectra were fitted by Shirley background subtraction before Gaussian–Lorentzian functions using CasaXPS software (Casa Software Ltd.).

The room-temperature Raman spectra were taken with a Renishaw, inVia confocal spectrometer (United Kingdom). The parameters used for the Raman measurements were as follows: laser with a wavelength of 785 nm (2.33 eV), power of the laser beam of 2 mW, and spectral resolution of 2 cm^−1^. The spectra obtained after normalization were analyzed using OMNIC spectroscopy software.

Fourier transform infrared spectroscopy (FTIR) was performed using a Thermo Scientific Nicolet IN10 MX microscope (USA). The spectra were recorded in a KBr pellet using a microscope in a transmission mode. The spectra were collected at a resolution of 4 cm^−1^, and 64 scans were averaged to obtain a single spectrum.

^1^H NMR spectra were recorded on an Agilent VNMRS system operated at 500 MHz. Chemical shifts $\delta$ are given in ppm, referenced to the solvent peak of CDCl_3_, defined at $\delta$ = 7.26 or DMSO-*d*_*6*_, defined at $\delta$ = 2.50. The following abbreviations were used for multiplicities: d (doublet), m (multiplet).

Materials were pyrolyzed using a Carbolite Gero STF 16/180 + 3216 Controller tube furnace. The resins with or without the CNOs were pyrolyzed in a tube furnace at 800 °C for 3 h in an Ar atmosphere. Ramping and cooling down were carried out in an Ar atmosphere as well, and the rate of both heating and cooling was 10 °C min^−1^.

To determine the pore size distributions of the materials low-temperature (77 K) N_2_ adsorption–desorption isotherms were measured using ASAP 2020 (Micromeritics, USA). Next, the Brunauer–Emmett–Teller (BET) surface area (*S*_*BET*_) using the appropriate model^57^ together with the procedure proposed by Rouquerol et al. was calculated^58^. Pore size distribution (PSD) curves were calculated via the nonlocal Density Functional Theory (DFT) for slit-like carbon pores^59–61^.

The PGSTAT 302N potentiostat (Autolab B.V., Metrohm, Utrecht, the Netherlands) connected with a three-electrode system consisting of a glassy carbon electrode (GCE, 10 × 2 mm) used as a working electrode, a Ag/AgCl reference electrode and an auxiliary Pt mesh electrode were used in cyclic voltammetry (CV) measurements. For the galvanostatic charge/discharge (GCD) tests, a symmetrical configuration of two identically modified GCE electrodes was used to ensure the most reliable results. Before starting the measurements, the surface of the GCE was modified by deposition of 15 µL of a dispersion containing the synthesized material in EtOH (3 mg mL^−1^) with the addition of a small amount of conductive carbon paint (CP, SPI Supplies, USA). Then the solvent was evaporated at RT. The electrolyte used in all electrochemical analyzes was 0.1 M KOH solution.

### Synthetic procedures

#### Synthesis of functionalized carbon nano-onions (f-CNO)

The CNO nanoparticles were dried in an oven (120 °C) overnight. Next, CNO (50 mg) was sonicated in anhydrous chlorobenzene (10 mL) under an Ar atmosphere for 30 min. *p*-Azidophenol (250 mg) was added, and the suspension was stirred for 24 h at 130 °C. After cooling, the reaction mixture was sonicated and centrifuged, followed by the separation of CNO from the dispersion. The purification process was repeated several times using DMF, toluene, and MeOH. The product was dried on a vacuum pump and then in a furnace (120 °C) to give 50 mg of the product as a black powder.

#### Synthesis of the composite containing resorcinol–formaldehyde resin and CNO (RF-CNO)

Na_2_CO_3_ (6 mg) was dissolved in HCHO (37 wt% in H_2_O; 1.13 g). *f*-CNO (15 mg) was added and the reaction mixture was sonicated for 5 min., followed by stirring for 30 min. at 25 °C. Resorcinol (1.1 g) was added and the reaction mixture was stirred for 1 h at 25 °C. Next, a solution of Pluronic F-127 (0.80 g) in H_2_O (4 mL) and EtOH (6 mL) was added, followed by the addition of 2 M HCl (1 mL). The reaction mixture was stirred for 1 h at 25 °C, affording the resin precipitation, and the reaction mixture was left without stirring overnight at the same temperature. The solution was then decanted and the gel was air-dried at 25 °C for 24 h and 80 °C for 24 h, resulting in 1.64 g of a black rigid gel.

#### Synthesis of the composite containing resorcinol–formaldehyde-melamine resin and CNO (RFM-CNO)

*f*-CNO (25 mg) was added to 0.05 M aqueous NaOH (1 mL). The dispersion was sonicated for 5 min., followed by stirring for 10 min. at 60 °C. Then, HCHO (37 wt% in H_2_O; 1.5 mL) and H_2_O (2.5 mL) were added to the reaction mixture and it was stirred for 30 min. at 25 °C. Next, resorcinol (0.991 g) and melamine (0.126 g) in 0.05 M NaOH (1.0 mL) were added and the reaction mixture was stirred for 10 min. at 60 °C. The resulting suspension was sealed in a glass tube and heated for 24 h at 50 °C in the furnace, followed by 5 days of heating at 80 °C. The resulting resin (black hydrogel) was purified for 3 days in acetone (replacing solvent every 8 h). The material was then filtered off and air-dried at 25 °C for 18 h, and then at 80 °C for 12 h, resulting in 1.38 g of a brown powder.

#### Synthesis of the composite containing benzoxazine resin and CNO (BX-CNO)

*f*-CNO (20 mg) was suspended in dioxane (1 mL), followed by sonification for 15 min. at RT. Then, HCHO (37 wt% in H_2_O; 0.1 g) was added, followed by sonification for 30 min. at RT. Next, the solution of bisphenol A (340 mg) in dioxane (2 mL) and HCHO (0.386 g) were added, followed by the dropwise addition of TETA (0.22 mL) maintaining the temperature below 10 °C. The reaction mixture was stirred for 1 h at RT. The resulting suspension was sealed in a glass tube and heated for 72 h at 80 °C in the furnace, affording a black hydrogel. Subsequent oven-drying at 80 °C afforded 2.80 g of a black product.

#### Synthesis of the composite containing calix[4]arene-based resin and CNO (CLX-CNO)

*f*-CNO (20 mg) was suspended in 5% aqueous NaOH (1.7 mL) and the suspension was sonicated for 30 min. at RT. Then, HCHO (37 wt% in H_2_O; 0.1 mL) was added and sonification was continued for 30 min. Next, phenylcalix[4]resorcinarene (0.92 mg) was dissolved in the solution of Pluronic F-127 (0.92 g) in 5% aqueous NaOH (5 mL) and EtOH (1.7 mL), and added to the CNO dispersion, followed by dropwise addition of HCHO (0.32 mL). The suspension was heated for 48 h at 90 °C, and the resulting precipitate was cooled down and washed with H_2_O. Next, it was dried at 100 °C for 10 h, affording ruby powder (1.55 g).

#### Synthesis of carbon materials (RF-CNO-C, RFM-CNO-C, BX-CNO-C, CLX-CNO-C)

The CNO-based resins were pyrolyzed in a tube furnace at 800 °C for 3 h in a stream of argon. Ramping and cooling down were carried out in a stream of argon as well, and the rate of both heating and cooling was 10 °C per minute.

## Results and discussion

### Synthesis of resins and carbon materials

**Figure 1:**
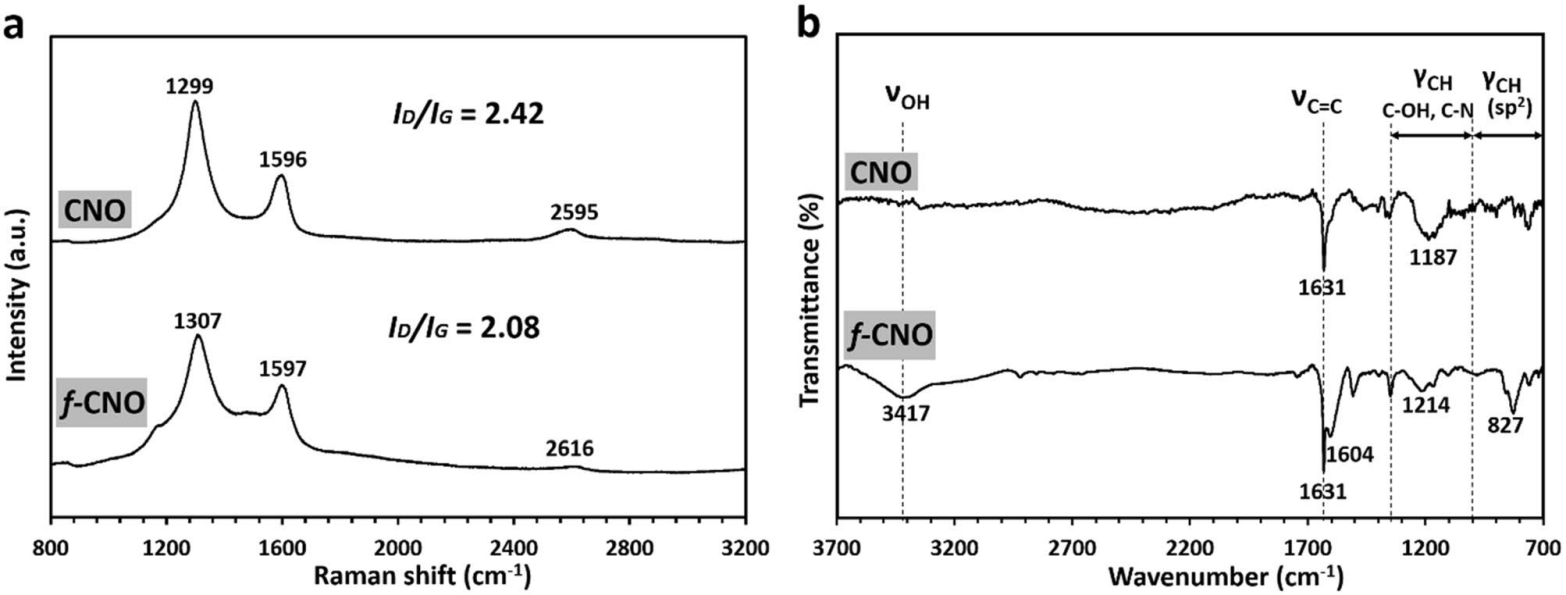
(**a**) Raman and (**b**) FTIR spectra of CNO and *f*-CNO.

The syntheses of resins hierarchically organized on the CNO surface were performed, forming a nanocarbon network. Initially, CNO was functionalized with the 4-hydroxyphenyl azide, affording *f*-CNO. This modification was confirmed by Raman spectroscopy (Fig. 1a). Generally, two “G” and “D” lines characterize carbon materials. For the single crystal of graphite, one line at 1580(± 5) cm^−1^ (“G” band) is observed^62^. A single line of the diamond at 1332 cm^−1^ (“D” band) is related to translational symmetry^63^. The ratio of intensities of these two bands (*I*_*D*_*/I*_*G*_) provides a quantitative description of the carbon microstructures (*e.g.*, crystalline order, in-plane crystal size, amount of sp^2^ or sp^3^ hybridized carbon atoms). For non-modified CNO, the G and D bands were observed at 1596 and 1299 cm^−1^, respectively (Fig. 1A). The additional peak at 2595 cm^−1^ corresponds to the second-order Raman spectrum, which is detected at approximately twice the wavenumber of the D band. For *f*-CNO, peaks assigned as D, G, and 2D were slightly shifted to higher wavenumbers, 1307, 1597 and 2616 cm^−1^, respectively.

The ratio of intensities between the D and G bands (*I*_*D*_/*I*_*G*_) depends mainly on the amount of sp^2^ and sp^3^ hybridized carbon atoms in CNs, which may be a confirming parameter of the covalent functionalization of the CNO^64^. In our case, the ratio of *I*_*D*_*/I*_*G*_ decreased from 2.42 for CNO to 2.08 for *f*-CNO, which is directly related to an increase of the sp^2^-hybridized carbon atoms number due to the formation of phenyl-substituted aziridine rings on the CNO surface. Furthermore, the FTIR analysis of the* f*-CNO (Fig. 1b) shows the characteristic stretching vibrations of -OH for Ar-OH moiety at 3417 cm^−1^ ^65^. The vibrations at 1214 cm^−1^ may indicate the presence of C-O or the formation of the C-N bond of the aziridine ring. The vibrations at 827 cm^−1^ can be assigned to new C-H bonds of Ar-OH on the modified CNO surface. The presence of Ar-OH moiety ensures the formation of a covalent linkage between the spherical CNO and the substrates involved in resin formation (phenolic condensation).

**Figure 2:**
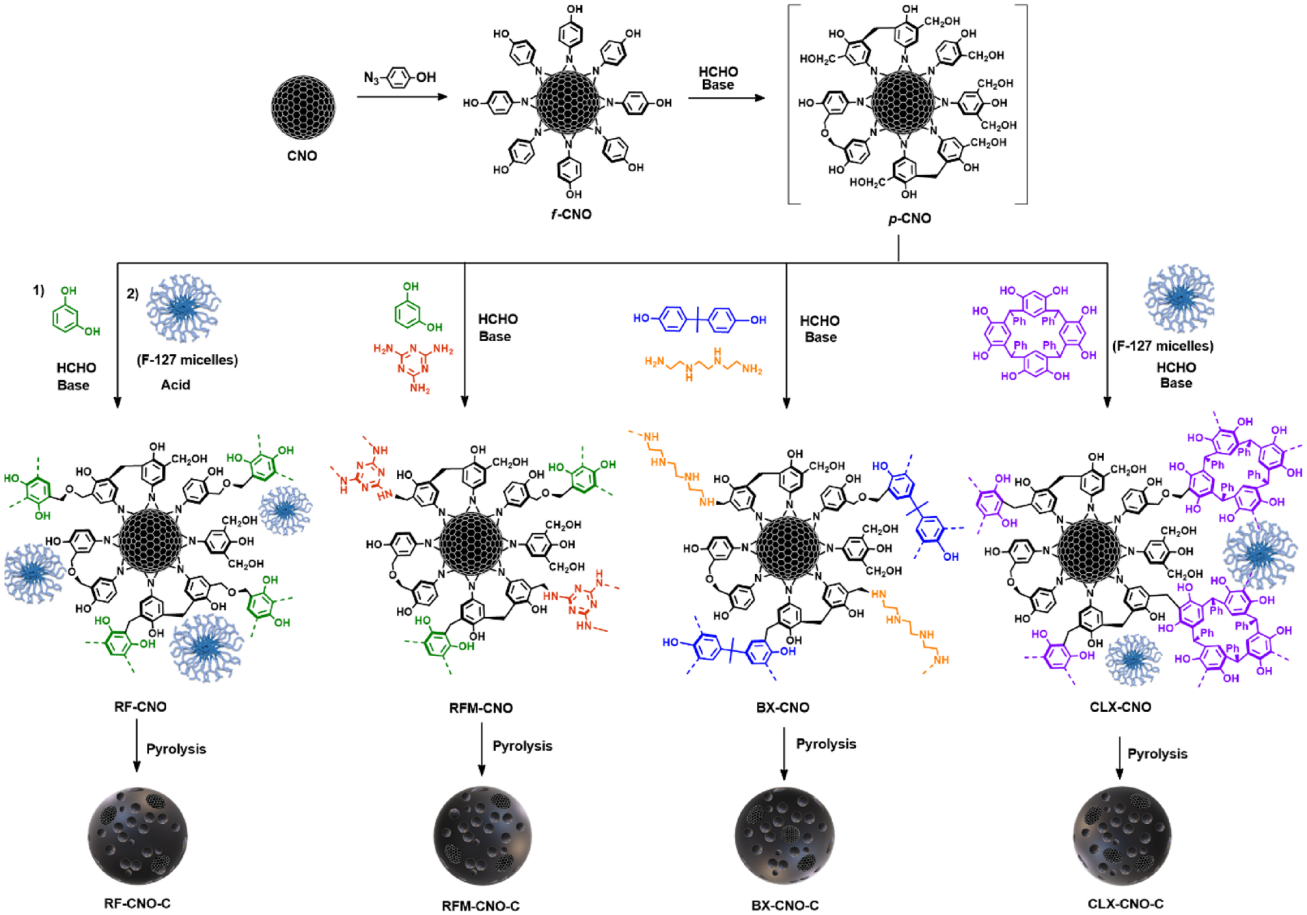
Representation of the CNO functionalization and synthesis of the composites containing CNO and resins.

Next, four monomers were used to synthesize the composites and additional carbon materials differing in N content. In some cases, an external porogenic agent (Pluronic F-127) was used. Each polymer synthesis was initiated by a hydro- or solvothermal reaction of the *f*-CNO with formaldehyde under primary conditions. This reaction guarantees the participation of the *f*-CNO in polycondensation reaction to form *p*-CNO, rather than simply agglomeration of the functionalized CNOs, which results in the lower reactivity of phenol compared to resorcinol derivatives. The condensation reactions of the *p*-CNO with various hydroxyaryl monomers and formaldehyde with or without amines led to the formation of several CNO composites containing (Fig. 2): (1) resorcinol–formaldehyde (**RF-CNO**), (2) resorcinol–formaldehyde-melamine (**RFM-CNO**), (3) benzoxazine (**BX-CNO**), and (4) calix[4]resorcinarene-derived resins (**CLX-CNO**). Subsequently, the composites were pyrolyzed at 800 °C for 3 h in an Ar atmosphere forming the carbon materials: **RF-CNO-C**, **RFM-CNO-C**, **BX-CNO-C**, and **CLX-CNO-C**. For comparison, subsequent reference polymeric materials (**RF**, **RFM**, **BX**, **CLX**) and carbons (**RF-C**, **RFM-C**, **BX-C**, **CLX-C**) were also synthesized (synthetic procedures, NMR and IR spectra of pristine resins before and after pyrolysis are given in SI). The weight loss of the material after pyrolysis was always more significant for the composites. The preliminary studies using pristine resins helped us calculate weight loss during pyrolysis and design experimental conditions for the syntheses of the composites, in which the CNO content was close to 5 wt%. Only in the case of **BX-CNO** the weight loss is almost four times higher than that, indicating the high impact of CNO presence by hindering the bond's formation, thus affording a more thermally unstable structure.

### Structure of resins and carbon materials

**Figure 3:**
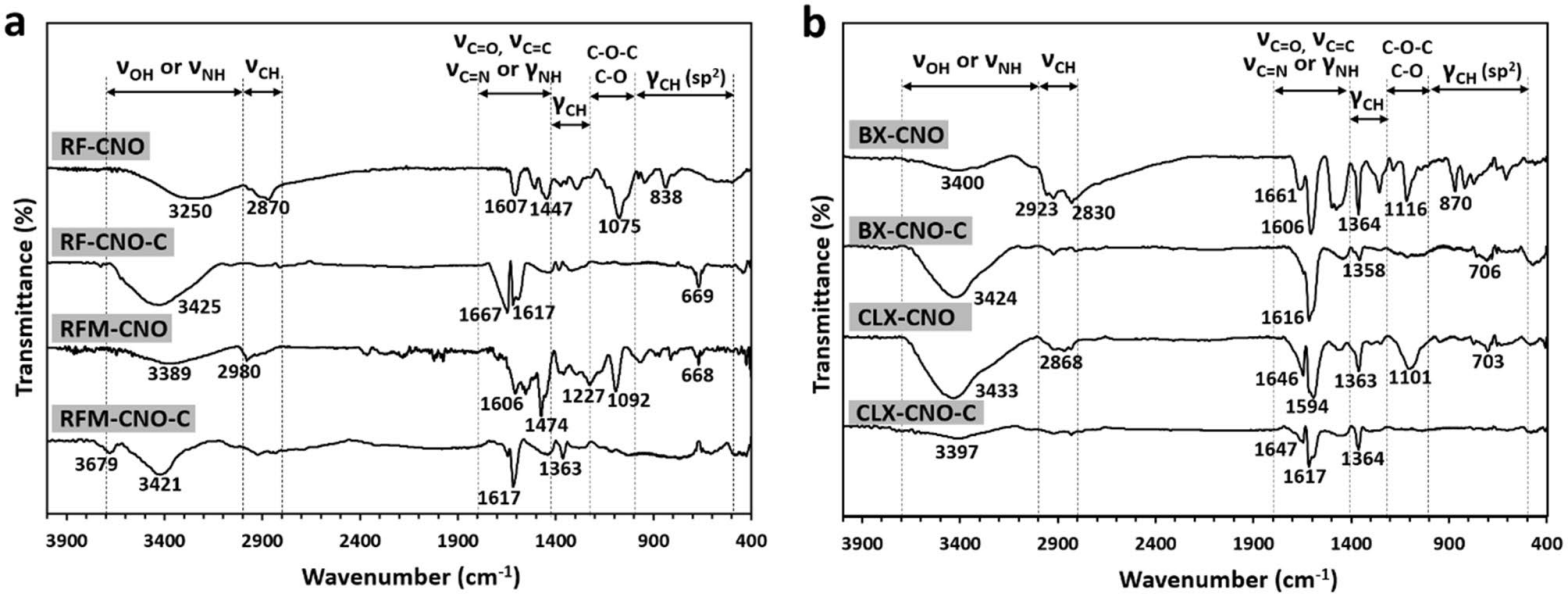
FTIR spectra of (**a**) **RF-CNO**, **RF-CNO-C**, **RFM-CNO**, **RFM-CNO-C**, and (**b**) **BX-CNO**, **BX-CNO-C**, **CLX-CNO**, **CLX-CNO-C**.

All composites and the carbonaceous materials were analyzed by FTIR spectroscopy (Fig. 3). **RF-CNO** is characterized mainly by bands at 3250, 2870, 1607, 1447, 1075, and 838 cm^−1^, which can be assigned to stretching vibrations of -OH, vibrations of the C=O and C=C groups, bending vibrations of C-H (sp^3^), presence of the ether and phenyl moieties, and vibrations of C-H in the aromatic ring, respectively (Fig. 3a). After pyrolysis of the composite giving **RF-CNO-C**, the spectrum is mainly dominated by vibrations at 3425, 1667, 1617, and 669 cm^−1^, which can be assigned to the presence of the –OH, C=O, C=C, and C–H group (Fig. 3a). For **RFM-CNO** resin, which possesses many N atoms in the network, the vibrations at 3425 and 1227 cm^−1^ were detected beside the bands characteristic for **RF-CNO**. These peaks can be attributed to vibrations of the -NH or C=N groups (Fig. 3a). For **RFM-CNO-C**, the most intensive vibrations were observed at 3679, 3421, 1617, and 1363 cm^−1^, which can be attributed to the –NH, –OH, C=N, C=C, C=O, and –CH groups (Fig. 3a). The two strong signals located at 2830 and 1364 cm^−1^ attributed to the stretching and the bending vibrations of –CH are present in TETA (**BX-CNO**, Fig. 3b). These infrared absorption bands distinguish this composite from those previously discussed.

After pyrolysis of **BX-CNO**, spectral analysis of **BX-CNO-C** shows the bands at 3424, 1616, 1358, and 706 cm^−1^, which indicate the presence of the –OH, C=C, –CH (sp^2^ and sp^3^) groups, that were the most thermally stable (Fig. 3b). The spectrum resembles the **RM-CNO** composite for the **CLX-CNO**, except for the bands at 1363 and 703 cm^−1^, which may be assigned to the –CH (sp^2^ and sp^3^) vibrations (Fig. 3b). Pyrolyzed material (**CLX-CNO-C**) possesses vibrations at 3397, 1647, 1617, and 1364 cm^−1^, typical for the carbonaceous skeleton containing the –OH, C=C, C=O, and –CH groups (Fig. 3b).

**Figure 4:**
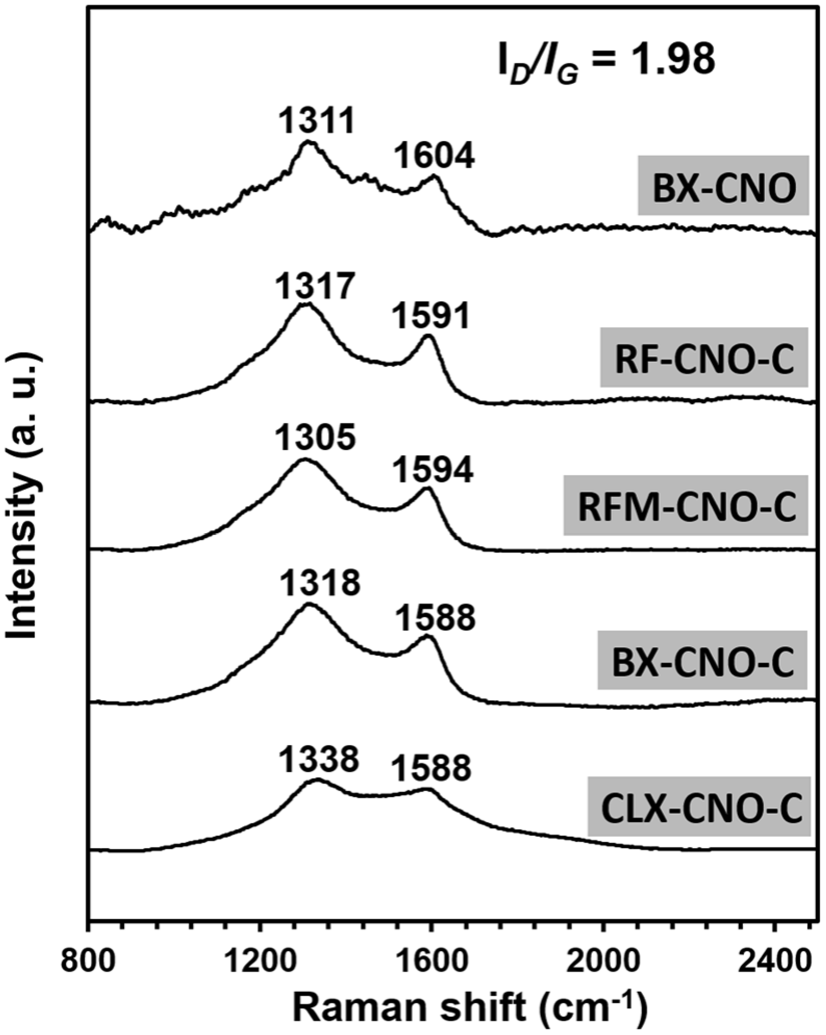
Raman spectra of **BX-CNO**, **RF-CNO-C**, **RFM-CNO-C**, **BX-CNO-C**, and **CLX-CNO-C**.

Raman spectroscopy was applied to analyze the composites containing CNO and polymers and their derived carbon materials (Fig. 4). The presence of the CN disorder induced the D and G bands characteristic for the CNO structure. These bands indicate the presence of the CNO in the composite and are dependent on the CNO wt% (Table 1).

**Table 1:** The compositions of the composite and the textural parameters determined by adsorption–desorption of N_2_.

Material	Residual mass (%)^a^	wt% CNO^b^	*S*_*BET*_ (m^2^ g^−1^)	Micropore volume (cm^3^ g^−1^)	Mesopore volume (cm^3^ g^−1^)	Total pore volume (cm^3^ g^−1^)
RF-C	21.8	–	688	0.244	0.317	0.561
RF-CNO-C	24.6	3.7	723	0.253	0.406	0.659
RFM-C	51.1	–	647	0.203	0.729	0.932
RFM-CNO-C	37.9	5.6	923	0.269	0.973	1.242
BX-C	34.0	–	–	–	–	–
BX-CNO-C	4.0	17.9	–	–	–	–
CLX-C	27.7	–	–	–	–	–
CLX-CNO-C	24.7	5.2	–	–	–	–

^a^Residual mass (%) = (*m*_*p*_:*m*_*C*_)·100%, where *m*_*p*_ is mass of the material obtained by pyrolysis, *m*_*c*_ is mass of the polymer or composite before pyrolysis.

^b^wt% CNO = (*m*_*CNO*_:*m*_*p*_)·100%, where *m*_*CNO*_ is the mass of CNOs subjected to the synthesis, *m*_*p*_ is the mass of pyrolyzed material.

Among polymers before pyrolysis, the D and G bands were noted only for **BX-CNO**, possessing the highest content of CNO in the material (CNO wt% = 17.9%) (Fig. 4). The *I*_*D*_*/I*_*G*_ intensity ratio of **BX-CNO** is equal to 1.98 and lower than the reference *f*-CNO (*I*_*D*_*/I*_*G*_ = 2.08; Fig. 1a). It indicates that due to the polycondensation reaction, the number of aromatic bonds of *f*-CNO further increases, which results in a decrease in the *I*_*D*_*/I*_*G*_ ratio. All carbon materials after pyrolysis (**RF-CNO-C**, **RFM-CNO-C**, **BX-CNO-C**, and **CLX-CNO-C**) possess wide overlapping bands at ca. 1315 and 1590 cm^−1^ (Fig. 4), which may be assigned to carbon atoms with sp^3^ and sp^2^ hybridization, respectively. These vibrations are characteristic of the carbon materials synthesized by transforming organic polymers into the carbonaceous skeleton^66^. As a result of the overlapping of these vibrations with the D and G bands of CNO, the CNO presence in the structure of the pyrolyzed composites cannot be unequivocally confirmed.

**Figure 5:**
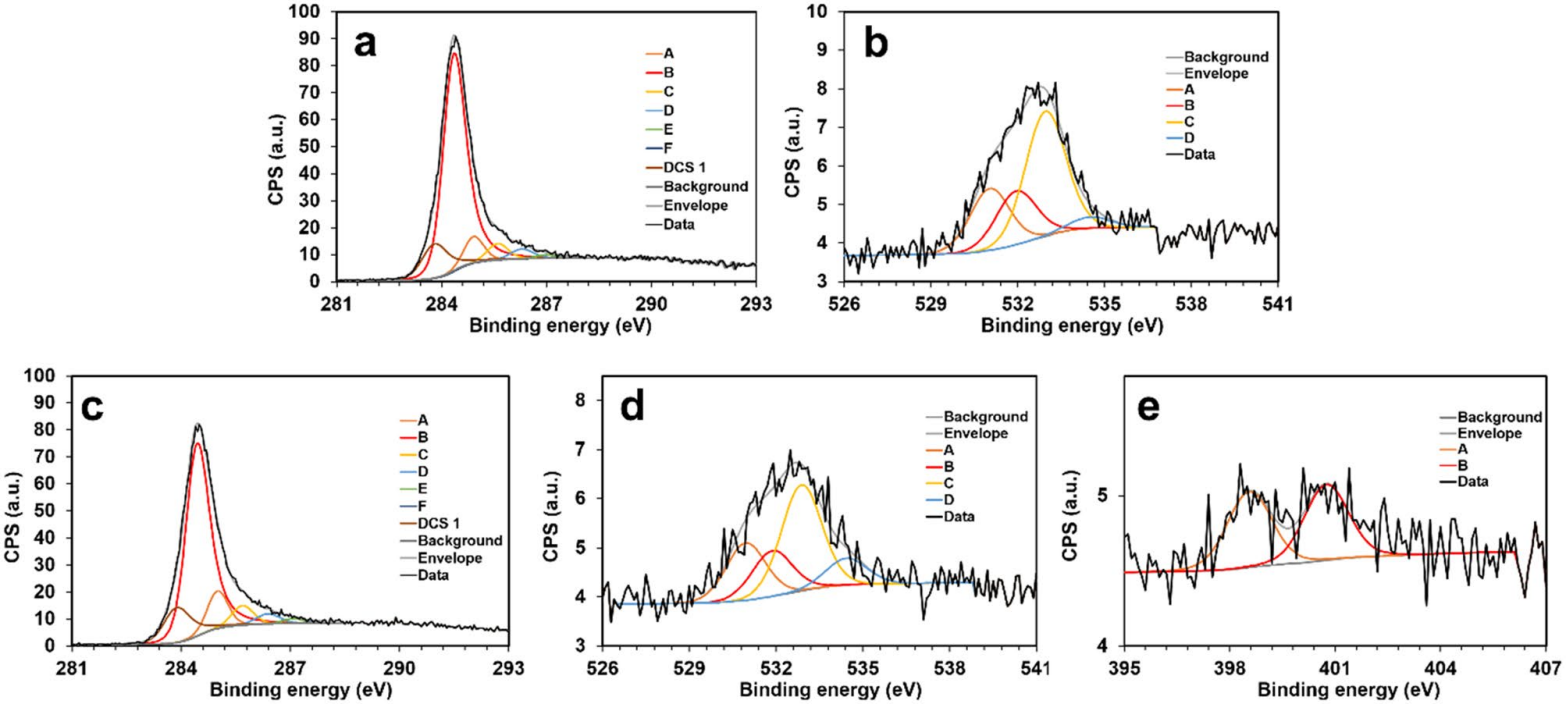
XPS spectra of the (**a**) **RF-CNO-C** (C 1 s), (**b**) **RF-CNO-C** (O 1 s), (**c**) **RFM-CNO-C** (C 1 s), (**d**) **RFM-CNO-C** (O 1 s), (**e**) **RFM-CNO-C** (N 1 s).

To describe the elemental surface composition of all CNO-derived materials (**RF-CNO-C**, **RFM-CNO-C**, **BX-CNO-C**, **CLX-CNO-C**) X-ray photoelectron spectroscopy (XPS) was used (Table 2). The distribution of elements was defined due to the deconvolution of the high-resolution spectral regions C1s, O1s, and N1s (Fig. 5 and Figure S4, SI). The percentages and assignments of species are given in Table 2, while the details of curve fitting are summarized in Tables S2–S4, SI. The results indicate that all studied materials contain carbon atoms in the amount of 85–92% and oxygen in the amount of 6 to 13%. The **RFM-CNO-C**, **BX-CNO-C**, and **CLX-CNO-C** materials contain N atoms (2–4%, Table S1, SI), which is related to the presence of this element in the structure of the *f*-CNO and the resin’s substrates. All materials have a similar pattern of distribution of functional groups and defects. The peak at ∼ 284.3 eV can be assigned to the sp^2^ hybridized C atom. The peaks at ∼ 284.9, ∼ 285.6, and ∼ 286.3 eV are related to the C–H sp^3^, C–C sp^3^, and C–OH or C–N moieties. The structures also contain negligible ether (∼ 287.0 eV) and carbonyl groups (∼ 287.8 eV). Interestingly, there are many vacancy-like defects in the graphitic lattice (∼ 283.8 eV) equal to 12–14% (Table 2 and Table S2, SI).

**Table 2:** Distribution of elements obtained from the deconvolution of the C1s, O1s, and N1s spectra by XPS.

Region	Peak	BE (eV)	Assignment	Ref.	Concentration (%)			
					RF-CNO-C	RFM-CNO-C	BX-CNO-C	CLX-CNO-C
C1s	A	284.9 ± 0.1	C–H sp^3^	^67^	7.1	10.4	16.8	10.9
	B	284.3 ± 0.1	C=C sp^2^	^68,69^	71.2	65.7	51.2	63.0
	C	285.6 ± 0.1	C–C sp^3^	^68,69^	4.5	5.7	10.4	7.0
	D	286.3 ± 0.1	C–OH, C–N	^68,69^	3.0	3.2	5.8	4.1
	E	287.0 ± 0.1	C–O–C	^68,69^	1.0	1.3	1.7	1.9
	F	287.8 ± 0.1	C=O	^68,69^	0.5	0.4	1.5	1.5
	DCS	283.8 ± 0.1	Defects in carbon structure	^70^	12.8	13.5	12.6	11.7
O1s	A	531.1 ± 0.1	C=O	^71^	24.3	24.0	14.7	29.1
	B	531.7 ± 0.3	C–OH	^72,73^	21.5	19.4	16.3	19.5
	C	532.7 ± 0.3	C–O–C, epoxy	^72,73^	49.7	45.0	45.5	38.9
	D	534.2 ± 0.3	Ph–OH	^71^	4.5	11.5	23.6	12.5
N1s	A	398.4 ± 0.2	–NH_2_	^74^	–	50.3	52.1	40.5
	B	400.7 ± 0.1	Protonated amine	^75^	–	49.7	48.0	24.1
	C	399.6 ± 0.1	Imine	^74^	–	–	–	35.4

An O1s XPS analysis shows that the ether and epoxy groups (∼532.7 eV) are dominant in **RF-CNO-C**, **RFM-CNO-C**, **BX-CNO-C**, and **CLX-CNO-C** materials. Furthermore, there are numerous carbonyl (∼531.1 eV), hydroxy (∼ 531.7 eV), and phenol (∼ 534.2 eV) groups in various proportions (Table S3, SI). The deconvolution of N1s XPS spectral regions of **RFM-CNO-C** and **BX-CNO-C** indicates the presence of NH_2_ (∼ 398.4 eV) and protonated amine (∼ 400.7 eV) in equal proportions. The XPS spectrum for **CLX-CNO-C** can be resolved into three regions corresponding to the NH_2_ (∼ 398.4 eV), protonated amine (∼ 400.7 eV), and imine groups (∼ 399.6 eV) (Table S4, SI).

**Figure 6:**
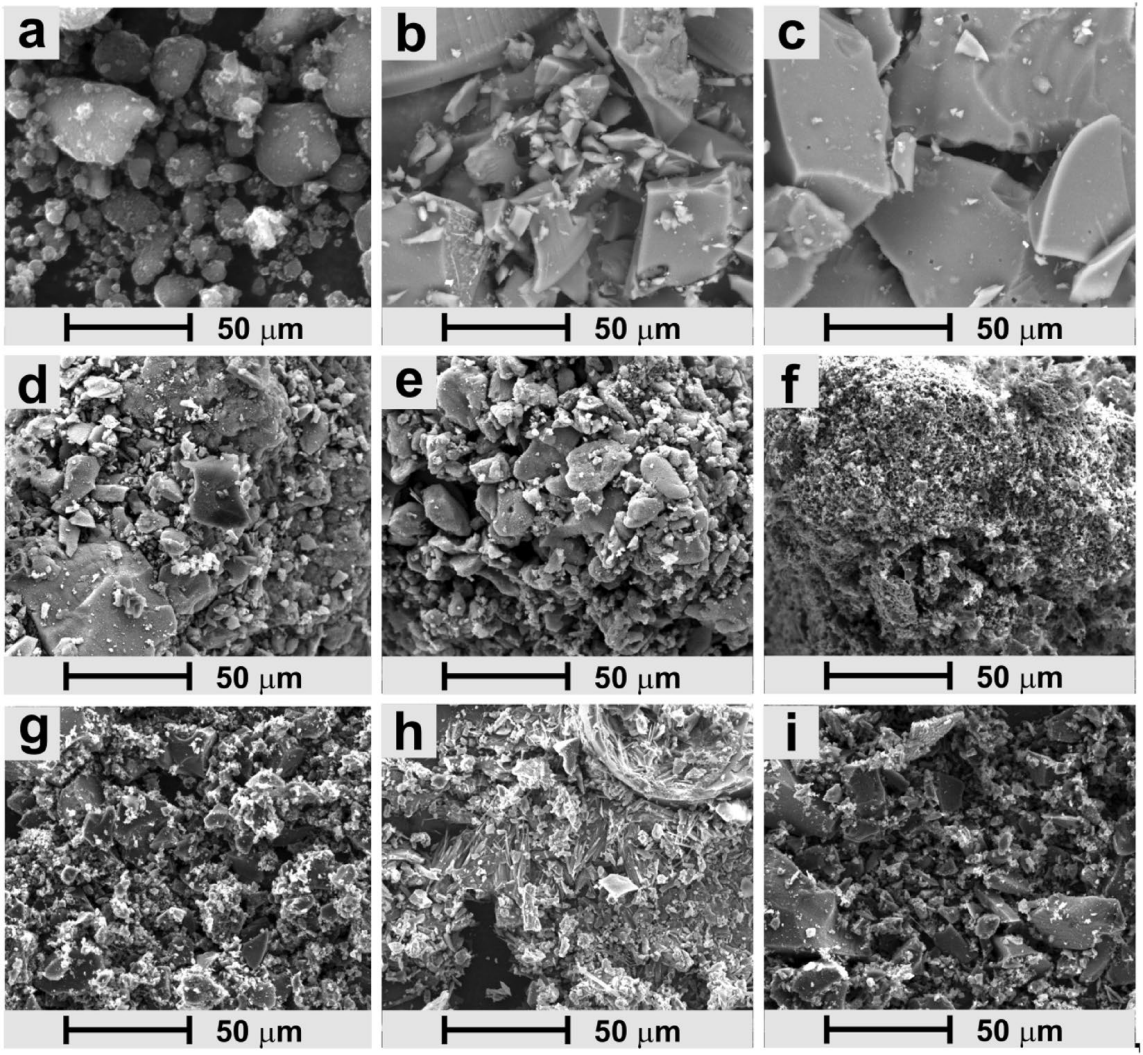
SEM images of (**a**) CNO, (**b**) **RF-C**, (c) **RF-CNO-C**, (**d**) **RFM-C**, (**e**) **RFM-CNO-C**, (**f**) **BX-C**, (**g**) **BX-CNO-C**, (**h**) **CLX-C**, and (**i**) **CLX-CNO-C**.

The morphology of selected materials was studied by scanning electron microscopy (SEM) (Fig. 6). CNO forms aggregates of spherical shape, varying in size from several hundred nanometers to several tens of micrometers. The surface of these forms is not smooth but porous, which is characteristic of carbon nanostructures (Fig. 6a). Polymeric materials create an entirely different structure. A significant difference can be seen in the case of **RF-C** and **RF-CNO-C** (Fig. 6b and Figure S4a, SI). Both materials have a smooth surface of the large aggregates. The composites containing CNO, **RF-CNO**, and **RF-CNO-C**, have a smooth, uniform texture with some macropores (Fig. 6c and Figure S4b, SI). Similarly, **RFM** and **RFM-C** structures have a uniform, smooth surface without pores marked (Fig. 6d and Figure S4c, SI). The **RFM-CNO** and **RFM-CNO-C** materials have patchy, heterogenous appearances with visible pores (Fig. 6e and Figure S4d, SI). The most porous morphology was observed for **BX-C** (Fig. 6f), which has a spongy granular structure. In the case of pyrolyzed CNO-based benzoxazine resin (**BX-CNO-C**), some aggregation of the structure was observed (Fig. 6g). The **CLX**-based material forms the needle- and flake-like structures (Fig. 6h), and after the addition of the CNO and further pyrolysis (**CLX-CNO-C**), it became more compact and less porous (Fig. 6i).

**Figure 7:**
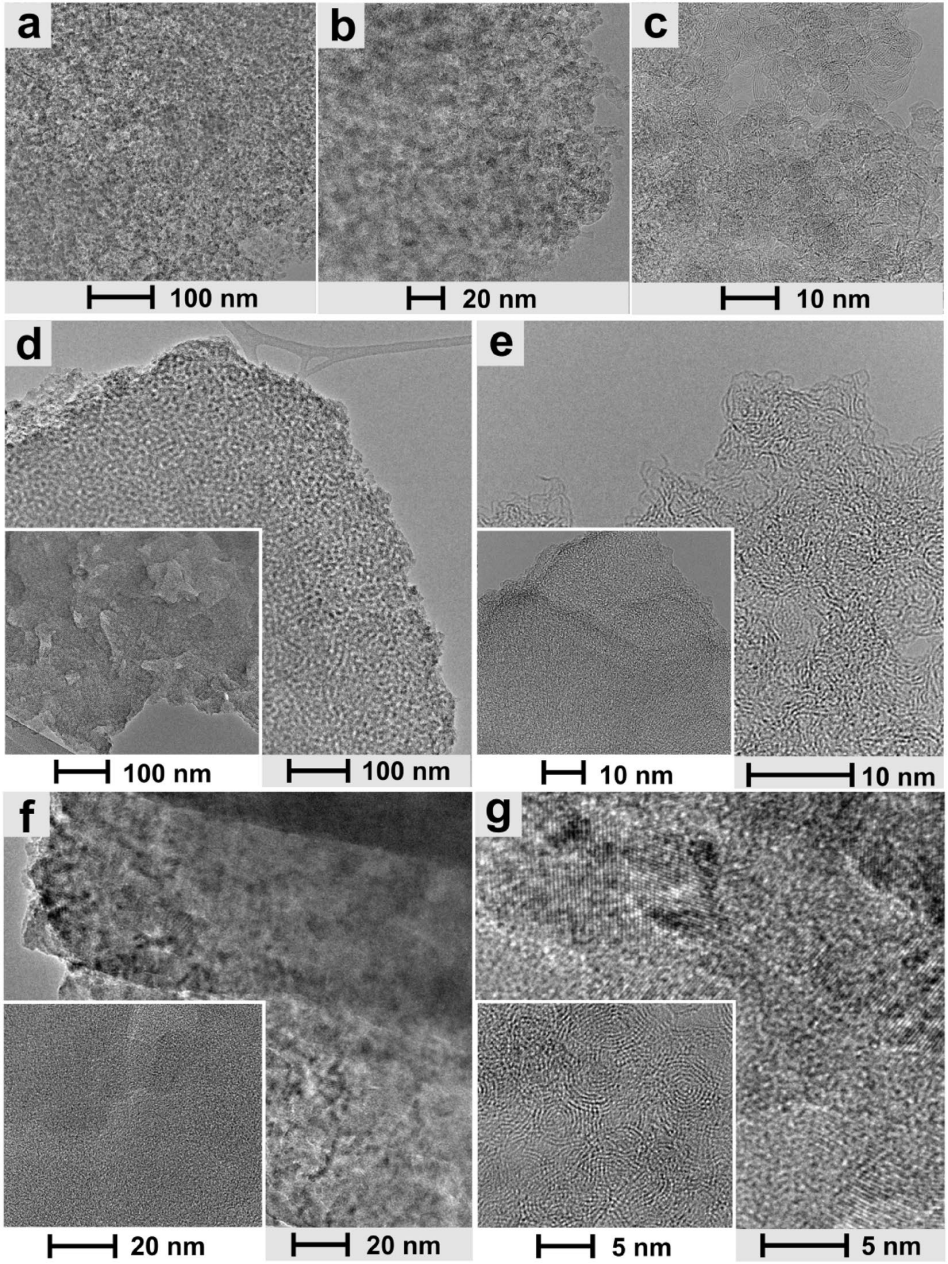
HRTEM images of (**a**–**c**) pristine CNO, (**d**, **e**) **RF-CNO-C** (inset: pristine RF-C), (**f**, **g**) **RFM-CNO-C** (insets: (**f**) pristine **RFM-C** and (**g**) pristine CNO) at the different magnifications.

Furthermore, high-resolution transmission electron microscopy (HRTEM) studies were performed to get additional structural information at the nanoscale (Fig. 7). Although all materials exhibit a compact homogeneous aggregate structure (Fig. 7d–g), structural differences are revealed when comparing composites with the pristine CNO (Fig. 7a–c and inset of Fig. 7d) or polymeric materials (insets of Fig. 7d–f).

The HRTEM image of **RF-CNO-C** proves the homogenous structure of the materials (Fig. 7d,e). The polymeric RF chains create poorly ordered domains on the CNO’s surface, on which small areas are visible, for which the parallel arrangement of polymer chains is found (Fig. 7e). Although the spherical CNO nanostructures are poorly visible, in the lower-resolution images (Fig. 7d), it is evident that the CNO is surrounded by a polymer, reproducing the spherical structure of the CN (Fig. 7a).

For comparison, we presented pristine **RF-C** (insets of Fig. 7d,e), where it is visible that the polymer itself creates an amorphous structure throughout the volume. Also, in the high-resolution image in Fig. 7e, the lack of amorphous structure in the composite indirectly confirms the presence of CNO in the entire material. The polymer chains forming on the surface of the CNO have a more ordered structure.

Besides the features mentioned above, the **RFM-CNO-C** composite creates a more orderly structure for which they stand out in amorphous and semicrystalline regions with well-marked ribbons (Fig. 7e). Again, when we compare the composite material with the pristine polymer **RFM-C**, we find that the material is ordered when CNO is added. Pristine polymer is amorphous, and the composite has distinct areas where spherical aggregates form (Fig. 7f), formed by CNOs surrounded by the polymer. For the higher magnification of TEM, large spaces with semicrystalline properties resembling diamond domains were found in the structure (Fig. 7g). Although the TEM images do not show CNO structures with distinct spherical graphene layers, it should be remembered that **RF** or **RMF** polymerization is carried out on the CNO surface. Therefore, composites have a system similar to a 'core–shell'; the 'core' is CNO, and the 'shell' is made of a polymer layer.

**Figure 8:**
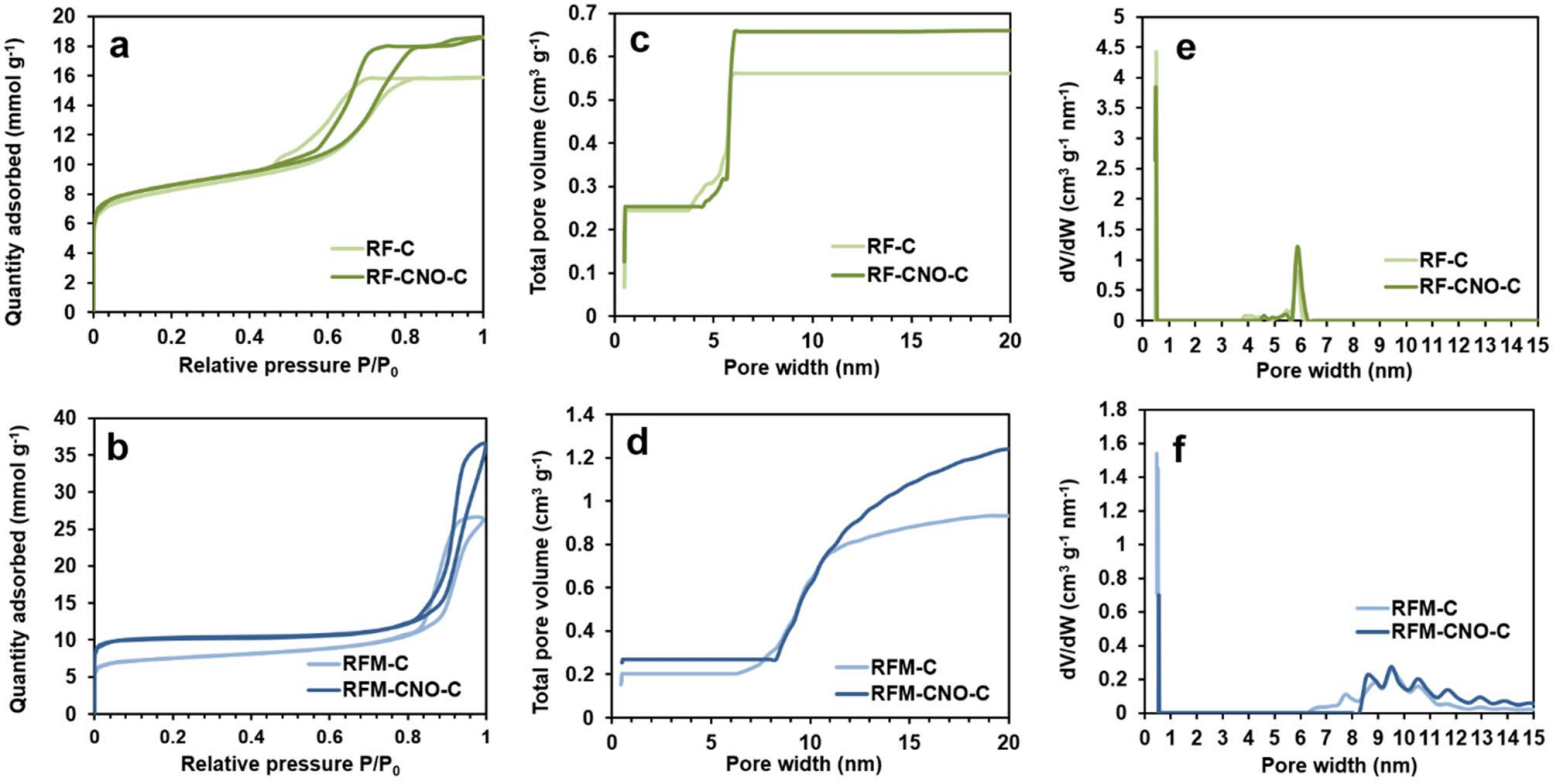
(**a**, **b**) N_2_ adsorption–desorption isotherms and (**c**, **d**) cumulative and (**e**, **f**) differential PSD of **RF-C**, **RF-CNO-C**, **RFM-C**, and **RFM-CNO-C** materials.

The textural parameters calculated from the N_2_ adsorption–desorption data are summarized in Table 1. At the same time, the N_2_ adsorption–desorption isotherms and the cumulative and differential pore size distribution (PSD) curves are shown in Fig. 8. The **RF-C**, **RF-CNO-C**, **RFM-C**, and **RFM-CNO-C** materials are porous, while no significant adsorption was observed for **BX-C**, **BX-CNO-C***,* **CLX-C**, and **CLX-CNO-C**. The Brunauer–Emmett–Teller surface area (*S*_*BET*_) values for **RF-C** and **RFM-C** materials are similar and equal to 688 and 647 m^2^ g^−1^, respectively (Table 1). The addition of CNO to the resin’s matrix increases the *S*_*BET*_ value to 723 m^2^ g^−1^ (**RF-CNO-C**) and 923 m^2^ g^−1^ (**RFM-CNO-C**).

The N_2_ adsorption–desorption curves recorded for all porous materials (**RF-C**, **RF-CNO-C**, **RFM-C**, and **RFM-CNO-C**) are of type IV, according to the IUPAC classification (Fig. 8a,b) that indicates the coexistence of micropores and mesopores in the structure of the analyzed materials^76^. Adsorbents characterized by a bimodal texture are regarded as hierarchical porous materials^77^. The plateau for **RF**- and **RFM**-based carbon materials observed at low pore width values indicate the homogeneous distribution of micropores in these systems (Fig. 8c,d). On the other hand, a plateau at higher pore width values, confirming a homogeneous distribution of mesopores, can only be observed in the case of material based on **RF** (in the range of 6–20 nm, Fig. 8c). The total pore volumes for **RF-C** and **RFM-C** were calculated to be 0.561 and 0.932 cm^3^ g^−1^, respectively (Table 1).

For both types of materials, the mesopore volume predominates, but in the case of **RF-C**, it is a slight predominance, while in the case of **RFM-C**, it is more than three times higher value (Table 1). Interestingly, the CNO addition to the materials increases the total pore volumes remarkably (0.659 cm^3^ g^−1^ for **RF-CNO-C** and 1.242 cm^3^ g^−1^ for **RFM-CNO-C**). Both materials' micropore and mesopore volumes rise, but the mesopore volume increase is more significant. The CNO addition to the carbon matrix slightly influences the pore width (Fig. 8e,f). The **RF-C**, **RF-CNO-C**, **RFM-C**, and **RFM-CNO-C** materials have micropores of a diameter of ca. 0.5 nm in a narrow range. The majority of mesopores of both **RF-C** and **RF-CNO-C** materials are in the limited range of c.a. 5.5–6.5 nm.

In contrast, the **RFM-C** and **RFM-CNO-C** possess the most larger mesopores in the diameter range of 8–11 nm. In the case of both RF and RFM systems, the CNO presence causes a slight shift of mesopore width to the higher values. In conclusion, **RFM-CNO-C** has the largest surface area and pore volume values with a predominance of mesopores. These textural properties should affect the electrochemical performance of **RFM-CNO-C**.

### Electrochemical performance of the hierarchical porous materials

**Figure 9:**
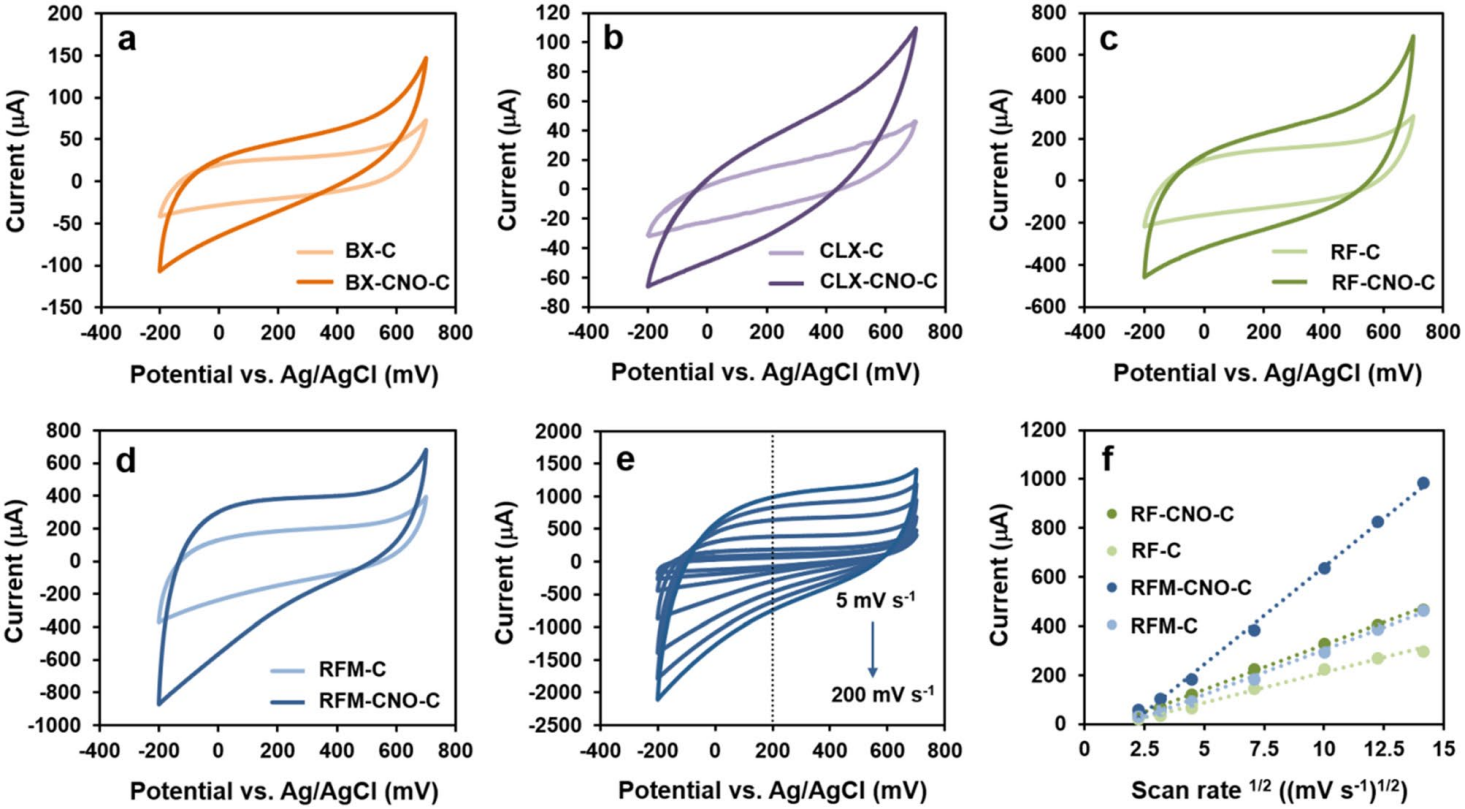
CV measurements were performed in a three-electrode configuration. (**a**–**d**) CV curves of all synthesized materials after pyrolysis recorded in 0.1 M KOH at 50 mV s^−1^. (**e**) CV curves recorded at the different scan rates for **RMF-CNO-C**. (**f**) Dependence of the current vs. the square root of the scan rate for the selected materials.

Cyclic voltammetry (CV) measurements were carried out with the working electrode modified with the pyrolyzed materials with a potential range from -200 to 700 mV (vs. Ag/AgCl) at a sweep rate of 50 mV s^−1^, which is shown in Fig. 9a–d. The quasi-rectangular shape of the CV curves indicates the capacitive nature of the studied materials resulting from the electrical double layer (EDL) charging process^25,78^. A much higher charge transfer resistance was observed for **CLX-C** and **CLX-CNO-C** than in the other materials (Fig. 9b). The **BX-C** and **BX-CNO-C** materials also exhibited lower capacitive current values (Fig. 9a).

**Figure 10:**
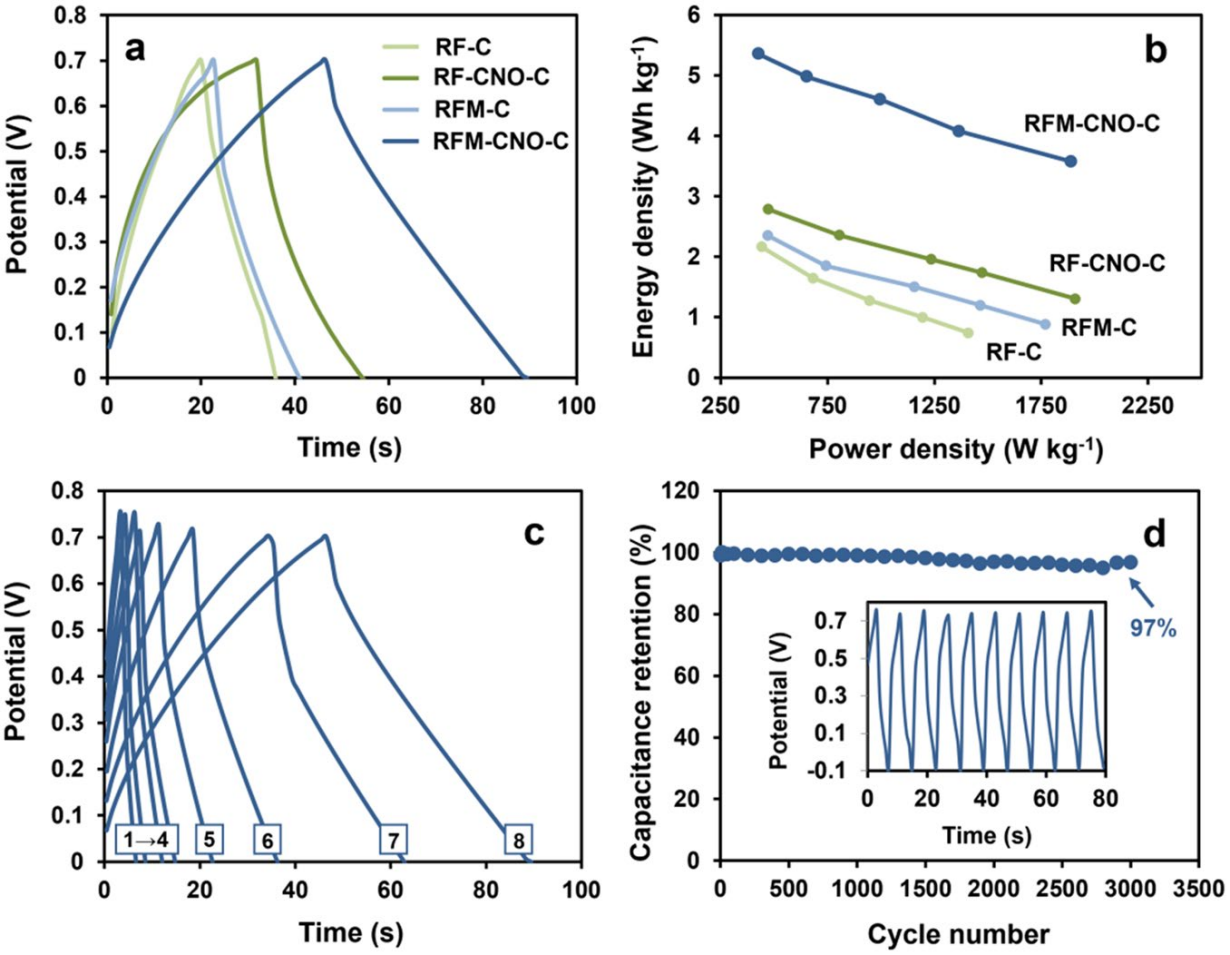
GCD measurements were performed in a two-electrode configuration. (**a**) GCD curves of selected materials recorded in 0.1 M KOH at the current density of 2 A g^−1^. (**b**) Ragone plots for selected materials. (**c**) GCD curves of **RFM-CNO-C** recorded in 0.1 M KOH at the different current densities: (1) 9; (2) 8; (3) 7; (4) 6; (5) 5; (6) 4; (7) 3; and (8) 2 A g^−1^. (**d**) Capacitance retention as a function of cycle number for **RFM-CNO-C** in 0.1 M KOH at the current density of 9 A g^−1^. Inset: First 10 galvanostatic GCD cycles recorded for **RFM-CNO-C**.

For these two series of non-porous materials, which determined the *S*_*BET*_ value was close to zero, the *C*_*S*_ values were calculated based on Eq. (1), ranging from 10 to 34 F g^−1^. The *C*_*s*_ value was calculated based on the mass of the deposited material on the electrode surface, *m*, within the potential range*, ΔE* = *(V*_*2*_*-V*_*1*_*),* according to the following formula:

1 $$ C_{s} = \frac{{\int_{{V_{1} }}^{{V_{2} }} {i(V){\text{d}}V} }}{{2vm(V_{2} - V_{1} )}} $$

where *i* symbolizes current and *v* is scan rate. The CNO addition resulted in a slight increase in the electrochemical capacitance, which is essential information in the context of the potential use of the obtained composites in SCs (Figs. 9 and 10, Table 3). The CV measurements recorded the highest capacitive currents for the **RF-C**, **RF-CNO-C**, **RFM-C**, and **RFM-CNO-C** materials. The *C*_*S*_ values calculated using the CV method are 105, 165, 133, and 278 F g^−1^, respectively (Table 3). One aspect must be emphasized when analyzing the *C*_*s*_ values with BET (Table 3). Apart from the porosity, the *C*_*s*_ value is also influenced by the pseudocapacitance, which is frequently related to heteroatoms in the material. Comparing the **RF-C** material with **RMF-C**, the latter has N atoms in its backbone. Doping carbon materials with nitrogen atoms improves the surface polarization of the material, its conductivity and electrochemical activity significantly. Moreover, introducing nitrogen atoms often results in pseudocapacitance in addition to the existing double-layer capacitance characterizing the carbon material. An improvement in the wettability of the material is observed, which also increases the *C*_*s*_ value.

**Table 3:** Specific capacitance, energy and power density values calculated from the GCD studies.

Material	*S*_*BET*_ (m^2^ g^−1^)	*C*_*S*_^a^ (F g^−1^)	*C*_*S*_^b^ (F g^−1^)	*E*_*density*_^c^ (Wh kg^−1^)	*P*_*density*_^c^ (kW kg^−1^)
RF-C	688	105	54	2	0.44
RF-CNO-C	723	165	85	3	0.47
RFM-C	647	133	69	2	0.47
RFM-CNO-C	923	278	160	5	0.43

^a^Specific capacitance calculated from CV curves at the sweep rate of 50 mV s^−1^ (three-electrode configuration). Calculated based on Eq. (1).

^b^Specific capacitance calculated from GCD studies at the current density of 2 A g^−1^ and potential window from 0 to + 700 mV (two-electrode configuration). Calculated based on Eq. (2).

^c^*E*_*density*_ and *P*_*density*_ calculated from GCD studies at the current density of 2 A g^−1^.

An increase in the *C*_*s*_ value was also observed after the CNO addition. These results closely correlate to the textural properties calculated for the hierarchical porous materials, in which the CNO addition increased the total pore volume of the materials (Tables 1 and 3). The correlation between the scan rate (from 5 to 200 mV s^−1^) and the capacitive current was also investigated (Fig. 9f). The CV curves obtained for the selected **RFM-CNO-C** material are shown in Fig. 9e. The analysis was carried out at the potential of 200 mV vs. Ag/AgCl.

An increase in the scan rates does not affect the shape of the CV curves. The rectangular shape voltammograms were observed even at high scan rates suggesting rapid charging/discharging properties. The CV analysis shows a linear relationship between the capacitive current and the square root of the polarization rate, which led to the conclusion that the charge storage mechanism is a diffusion-controlled process (Fig. 9f).

Subsequently, the selected materials were subjected to galvanostatic charge–discharge (GCD) tests in two-electrode symmetrical configuration at different current densities in the range of 2–6 A g^−1^ (Fig. 10). The capacitances at different current densities were determined (Eq. 2) and are summarized in Table 3, and Table S5 in SI. The *C*_*s*_ value was calculated based on the mass of the deposited material on the electrode surface, *m*, within the potential range (Δ*V* = (*V*_*2*_–*V*_*1*_)), measuring the current value (*i*) and discharge time (*t*_*d*_), according to the following formula:

2 $$ C_{s} = \frac{{it_{d} }}{m\Delta V} $$

For all porous materials, high reversible specific capacitances were observed at 2 A g^−1^: **RF-C** of 54 F g^−1^, **RF-CNO-C** of 85 F g^−1^, **RFM-C** of 69 F g^−1^, and **RFM-CNO-C** of 160 F g^−1^. When the current densities increase to 6 A g^−1^, the *C*_*S*_ values for all porous materials decrease to c.a. 50% of their initial values. Moreover, the GCD cycles recorded in a wide potential window (from 0 to 700 mV) showed nearly symmetric triangular curves that prove the EDL capacitive nature of the studied materials. The slight deviations from the ideal triangular profile were observed for the lower current densities approaching the value of 2 A g^−1^, resulting from the difference in the micro and mesopores' participation in the electrode layer charging (Fig. 10a,c).

The values of energy density (*E*_*density*_) and power density (*P*_*density*_) determined on the GCD studies are summarized in Table 3, and the relationships between them are presented on a Ragone plot (Fig. 10b). The maximum *E*_*density*_ was 5 Wh kg^−1^ at a power density of 0.43 kWh kg^−1^ for **RFM-CNO-C**. For other materials, the *E*_*density*_ values varied from 1 to 4 Wh kg^−1^, with high, almost unchanged the *P*_*density*_ values ranging from 0.44 to 1.9 kWh kg^−1^. The *E*_*density*_ value was significantly increased, suggesting that **RFM-CNO-C** offers remarkable charge storage in an aqueous electrolyte. The **RFM-CNO-C** material was further studied at the different current densities ranging from 2 to 9 A g^−1^ (Fig. 10c), and its cycling stability at the current density of 9 A g^−1^ (Fig. 10d). **RFM-CNO-C** exhibited the *C*_*S*_ value of 160 F g^−1^ at a current density of 2 A g^−1^ that tends to be stable. After 3000 cycles, the **RFM-CNO-C** electrode maintains about 97% of its initial capacitive efficiency. The results revealed excellent cycling stability of the **RFM-CNO-C** electrode, which can be attributed to the stability of the hierarchical porosity.

The specific capacitance value of **RFM-CNO-C** is very high compared to other composites consisting of carbonized polymers and carbon nanostructures (Table S6, SI). Among the hybrid materials, the most commonly used is graphene coated with various polymer-derived materials. However, the amount of graphene in these materials is much more significant than the carbon nano-onion in **RFM-CNO-C** (5 wt% CNO). Moreover, adding only a tiny amount of CNO results in an improvement in electrochemical performance.

## Conclusions

In summary, we developed a series of hierarchical porous carbon materials derived from resins and spherical carbon nanostructures that have been prepared through a combination of condensation polymerization in the presence of CNO and pyrolysis. The CNO was covalently functionalized before the polycondensation reaction. The obtained carbon materials possess poorly ordered domains with some structural disorder. It must be highlighted that the **RFM-CNO-C** composite exhibits a more orderly structure in which amorphous and semicrystalline regions are present. Interestingly, the CNO addition to the materials remarkably increases the total pore volumes (to 0.932 cm^3^ g^−1^ for **RF-CNO-C** and 1.242 cm^3^ g^−1^ for **RFM-CNO-C**). The micropore and mesopore volumes rise in both materials showing the best textural performances, but the mesopore volume increase is more significant.

Depending on the porosity, pore volume, heteroatom content, and morphology, all nanostructured carbon exhibited good electrochemical performance. The carbon material derived from CNO, resorcinol, and melamine (**RFM-CNO-C**) showed the highest *C*_*S*_ value of 160 F g^−1^ at a current density of 2 A g^−1^ that tends to be stable after 3000 cycles. The **RFM-CNO-C** electrode maintains about 97% of its initial capacitive efficiency. The results revealed excellent cycling stability of the **RFM-CNO-C** electrode, which can be attributed to the strength of the hierarchical porosity owing to its unique structural features and good electrochemical properties, making it promising electrode material for SC devices.

## Supplementary Information

Supplementary Information 1.

Supplementary Information 2.

## Data Availability

The datasets used and analyzed during the current study available from the corresponding author on reasonable request.
