# Supplementary material for: Composites containing resins and carbon nano-onions as efficient porous carbon materials for supercapacitors
Source: Sci Rep. 2023 Apr 24;13:6606. doi: 10.1038/s41598-023-33874-w (PMC10126139; doi:10.1038/s41598-023-33874-w)
Supplement: Supplementary file 1 — Supplementary Information 1. [file 41598_2023_33874_MOESM1_ESM.docx]

**Supplementary Material**

Composites containing resins and carbon nano-onions as efficient porous carbon materials for supercapacitors

Gabriela Siemiaszko^1^*, Joanna Breczko^1,2^, Agnieszka Hryniewicka^1^, Anna Ilnicka^3^, Karolina H. Markiewicz^2^, Artur P. Terzyk^3^, Marta E. Plonska-Brzezinska^1^*

1. *Department of Organic Chemistry, Faculty of Pharmacy with the Division of Laboratory Medicine, Medical University of Bialystok, Mickiewicza 2A, 15-222 Bialystok, Poland*

*E-mail: gsiemiaszko@gmail.com, marta.plonska-brzezinska@umb.edu.pl*

1. *Faculty of Chemistry, University of Bialystok, Ciolkowskiego 1K, 15-245 Bialystok, Poland*
2. *Faculty of Chemistry, Nicolaus Copernicus University in Torun, Gagarina 7, 87-100 Torun, Poland*

Table of contents

[1. SYNTHETIC PROCEDURES 3](#_Toc124158240)

[1.1. Synthesis of *p*-azidophenol 3](#_Toc124158241)

[1.2. Synthesis of phenylcalix[4]resorcinarene 3](#_Toc124158242)

[1.3. Synthesis of resorcinol-formaldehyde resin (**RF**) 3](#_Toc124158243)

[1.4. Synthesis of benzoxazine resin (**RFM**) 4](#_Toc124158244)

[1.5. Synthesis of benzoxazine resin (**BX**) 4](#_Toc124158245)

[1.6. Synthesis of phenylcalix[4]resorcinarene-derived resin (**CLX**) 4](#_Toc124158246)

[1.7. Synthesis of carbon materials (**RF-C**, **RFM-C**, **BX-C**, **CLX-C**) 4](#_Toc124158247)

[2. TABLES AND FIGURES 5](#_Toc124158248)

[Table S1. Surface Elemental Composition of selected carbon samples determined by XPS. 5](#_Toc124158249)

[Table S2. Chemical state, positions, FWHM, and relative area percentages of the deconvoluted C 1s peaks obtained from XPS analyses of RF-CNO-C, RFM-CNO-C, BX-CNO-C, and CLX-CNO-C samples. 5](#_Toc124158250)

[Table S3. Chemical state, positions, FWHM, and relative area percentages of the deconvoluted O 1s peaks obtained from XPS analyses of RF-CNO-C, RFM-CNO-C, BX-CNO-C, and CLX-CNO-C samples. 6](#_Toc124158251)

[Table S4. Chemical state, positions, FWHM, and relative area percentages of the deconvoluted N 1s peaks obtained from XPS analyses of RF-CNO-C, RFM-CNO-C, BX-CNO-C, and CLX-CNO-C samples. 6](#_Toc124158252)

[Table S5. Specific capacitance, energy, and power values calculated from the GCD studies. 7](#_Toc124158253)

[Table 6. Comparison of the polymer-derived carbon nanostructure-based materials 8](#_Toc124158254)

[Figure S1. ^1^H NMR spectrum of *p*-azidophenol (500 MHz, CDCl_3_). 10](#_Toc124158255)

[Figure S2. ^1^H NMR spectrum of phenylcalix[4]resorcinarene (500 MHz, DMSO-*d_6_*). 10](#_Toc124158256)

[Figure S3. FTIR spectra of (a) RF, (b) RF-C, (c) RFM, (d) RFM-C, (e) BX, (f) BX-C, (g) CLX, (h) CLX-C. 11](#_Toc124158257)

[Figure S4. XPS spectra of the (A) BX-CNO-C and (B) CLX-CNO-C spectral regions (a) C 1s, (b) O1 s, and (c) N 1s. 11](#_Toc124158258)

[Figure S5. SEM images of (a) RF, (b) RF-CNO, (c) RFM, (d) RFM-CNO. 12](#_Toc124158259)

[References 12](#_Toc124158260)

# SYNTHETIC PROCEDURES

## Synthesis of *p*-azidophenol^1^

The solution of NaNO_2_ (1.5 equiv, 1.37 mmol, 94.8 mg) in H_2_O (3 mL) was added to *p*-aminophenol (1.0 equiv, 0.916 mmol, 100 mg) in 0.5 M HCl (4 mL) dropwise at 0 °C. Next, the solution of NaN_3_ (1.5 equiv, 1.37 mmol, 89.1 mg) in H_2_O (3 mL) was added dropwise, while maintaining the temperature at 0 °C. The reaction mixture was stirred at this temperature for 0.5 h and the full conversion of *p*-aminophenol was noted by TLC (eluent 1/1 hexanes/AcOEt + several drops of MeOH; visualized by UV or vanillin stain). The reaction mixture was extracted using AcOEt and DCM, respectively, dried over MgSO_4,_ and concentrated using a rotary evaporator. Purification by flash column chromatography on silica gel (eluent 8/2 hexanes/AcOEt) afforded a product as a brown liquid (51 mg, yield 41%). ^1^H NMR (500 MHz, CDCl_3_): *δ* 9.51 (s, 1H, OH), 6.86 (dd, *J* = 65.1, 8.8 Hz, 4H, Ar).

## Synthesis of phenylcalix[4]resorcinarene^2^

To resorcinol (1 equiv, 50 mmol, 5.5 g) and benzaldehyde (1 equiv, 50 mmol, 5.10 mL) in EtOH/H_2_O mixture (1:1, 20 mL) concentrated HCl (5 mL) was added dropwise. The reaction mixture was stirred for 2 h at 75 °C and the full conversion of substrates was noted by TLC (eluent 1/1 hexanes/AcOEt; visualized by Cerium Ammonium Molybdate stain). The precipitate was purified by excessive washing with H_2_O and oven-dried at 80 °C, affording 6.88 g of a product as a brownish-green powder (yield 66%). ^1^H NMR (500 MHz, DMSO-*d_6_*): *δ* 5.53-5.64 (m, 4H, ArCH), 6.13-6.95 (m, 28H, ArH), 8.44-8.54 (4H, OH).

## Synthesis of resorcinol-formaldehyde resin (RF)^3^

Na_2_CO_3_ (17 mg) was dissolved in HCHO (37 wt% in H_2_O; 3.39 g). Resorcinol (3.30 g) was added and the reaction mixture was stirred for 1 h at 25 °C. Next, a solution of Pluronic F-127 (2.40 g) in the EtOH/H_2_O mixture (2:1, 30 mL) was added, followed by the addition of 2 M HCl (3 mL). The reaction mixture was stirred for 1 h at 25 °C, affording the resin precipitation, and the reaction mixture was left without stirring overnight at the same temperature. The solution was then decanted and the gel was air-dried at 25 °C for 24 h and 80 °C for 24 h, resulting in 4.55 g of an orange rigid gel.

## Synthesis of benzoxazine resin (RFM)^4^

Resorcinol (1.98 g) and melamine (0.252 g) were added to 0.1 M aqueous NaOH (2 mL) and the reaction mixture was stirred for 10 min. at 60 °C. Next, formaldehyde (37 wt% in H_2_O; 3 mL) and water (7 mL) were added, followed by stirring for 30 min. at 25 °C. The resulting clear yellowish solution material was sealed in a glass tube and heated for 24 h at 50 °C in the furnace, followed by 5 days of heating at 80 °C. The resulting resin (maroon hydrogel) was purified for 3 days in acetone (replacing acetone every 8 h). The material was then filtered off and air-dried at 25 °C for 18 h, and then at 80 ° C for 12 h, resulting in 3.34 g of a brown powder.

## Synthesis of benzoxazine resin (BX)^5^

Bisphenol A (2.26 g) was dissolved in dioxane (10 mL). Next, formaldehyde (37 wt% in H_2_O; 3.24 g) was added, while maintaining the temperature below 10 °C. Subsequently, TETA (1.48 mL) was added dropwise and the reaction mixture was stirred for 1 h at room temperature. After that, the resulting solution was sealed in a glass tube and heated in a furnace at 80 °C for 72 h, affording the brown-yellow hydrogel. Subsequent oven-drying at 80 °C afforded 4.36 g of brown-yellow hard product.

## Synthesis of phenylcalix[4]resorcinarene-derived resin (CLX)^6^

Phenylcalix[4]resorcinarene (2.78 g) was dissolved in the solution of Pluronic F-127 (2.78 g) in 5% aqueous NaOH (20 mL) with EtOH (5 mL). Next, formaldehyde (37 wt% in H_2_O; 0.78 mL) was added dropwise to the reaction mixture and it was stirred for 20 h at 90 °C. The precipitate was filtered, washed with an excess of H_2_O, and oven-dried at 100 °C, affording 4.88 g of the product as a purple-black solid.

## Synthesis of carbon materials (RF-C, RFM-C, BX-C, CLX-C)

All resins (**RF**, **RFM**, **BX**, **CLX**) were pyrolyzed in a tube furnace at 800 °C for 3 hours in a stream of argon. Ramping and cooling down were carried out in a stream of argon as well, and the rate of both heating and cooling was 10 °C per minute.

# TABLES AND FIGURES

# Table S1. Surface Elemental Composition of selected carbon samples determined by XPS.

| Sample | Elements (%) | | |
| --- | --- | --- | --- |
|  | **C** | **N** | **O** |
| RF-CNO-C | 92.3 ± 2.0 | - | 7.7 ± 2.0 |
| RFM-CNO-C | 92.1 ± 2.1 | 2.3 ± 1.3 | 5.6 ± 1.8 |
| BX-CNO-C | 89.2 ± 2.2 | 4.2 ± 1.3 | 6.6 ± 1.9 |
| CLX-CNO-C | 84.6 ± 1.9 | 2.6 ± 1.2 | 12.8 ± 1.7 |

# Table S2. Chemical state, positions, FWHM, and relative area percentages of the deconvoluted C 1s peaks obtained from XPS analyses of RF-CNO-C, RFM-CNO-C, BX-CNO-C, and CLX-CNO-C samples.

| **Region** | **Species** | **RF-CNO-C** | | | **RFM-CNO-C** | | | **BX-CNO-C** | | | **CLX-CNO-C** | | |
| --- | --- | --- | --- | --- | --- | --- | --- | --- | --- | --- | --- | --- | --- |
|  |  | **Peak**  **(eV)** | **FWHM (eV)** | **% Area** | **Peak (eV)** | **FWHM (eV)** | **% Area** | **Peak (eV)** | **FWHM (eV)** | **% Area** | **Peak (eV)** | **FWHM (eV)** | **% Area** |
| **C 1s A** | C-H sp^3^ | 284.91 | 0.67 | 7.1 | 285.00 | 0.67 | 10.4 | 284.96 | 0.70 | 16.8 | 284.94 | 0.68 | 10.9 |
| **C 1s B** | C=C sp^2^ | 284.32 | 0.73 | 71.2 | 284.41 | 0.73 | 65.7 | 284.41 | 0.72 | 51.2 | 284.35 | 0.73 | 63.0 |
| **C 1s C** | C-C sp^3^ | 285.60 | 0.70 | 4.5 | 285.69 | 0.70 | 5.7 | 285.55 | 0.73 | 10.4 | 285.63 | 0.71 | 7.0 |
| **C 1s D** | C-OH, C-N | 286.30 | 0.78 | 3.0 | 286.38 | 0.77 | 3.2 | 286.28 | 0.81 | 5.8 | 286.32 | 0.78 | 4.1 |
| **C 1s E** | C-O-C | 286.99 | 0.68 | 1.0 | 287.08 | 0.67 | 1.3 | 287.04 | 0.68 | 1.7 | 287.02 | 0.68 | 1.9 |
| **C 1s F** | C=O | 287.77 | 0.70 | 0.5 | 287.86 | 0.68 | 0.4 | 287.81 | 0.71 | 1.5 | 287.80 | 0.71 | 1.5 |
| **C 1s DCS 1** | defects in the carbon structure | 283.76 | 0.83 | 12.8 | 283.84 | 0.83 | 13.5 | 283.88 | 0.83 | 12.6 | 283.78 | 0.83 | 11.7 |

# Table S3. Chemical state, positions, FWHM, and relative area percentages of the deconvoluted O 1s peaks obtained from XPS analyses of RF-CNO-C, RFM-CNO-C, BX-CNO-C, and CLX-CNO-C samples.

| **Region** | **Species** | **RF-CNO-C** | | | **RFM-CNO-C** | | | | **BX-CNO-C** | | | **CLX-CNO-C** | | |
| --- | --- | --- | --- | --- | --- | --- | --- | --- | --- | --- | --- | --- | --- | --- |
|  |  | **Peak**  **(eV)** | **FWHM (eV)** | **% Area** | **Peak (eV)** | **FWHM (eV)** | **% Area** | **Peak (eV)** | | **FWHM (eV)** | **% Area** | **Peak (eV)** | **FWHM (eV)** | **% Area** |
| **O 1s A** | C=O | 531.04 | 1.61 | 24.3 | 530.96 | 1.61 | 24.0 | 530.48 | | 1.61 | 14.7 | 530.84 | 1.61 | 29.1 |
| **O 1s B** | C-OH, C-O-C | 531.95 | 1.64 | 21.5 | 531.87 | 1.64 | 19.4 | 531.39 | | 1.64 | 16.3 | 531.75 | 1.64 | 19.5 |
| **O 1s C** | C-O-C, epoxy | 532.96 | 1.64 | 49.7 | 532.88 | 1.64 | 45.0 | 532.40 | | 1.64 | 45.5 | 532.76 | 1.64 | 38.9 |
| **O 1s D** | Ph-OH | 534.51 | 1.66 | 4.5 | 534.43 | 1.66 | 11.5 | 533.95 | | 1.66 | 23.6 | 534.31 | 1.66 | 12.5 |

# Table S4. Chemical state, positions, FWHM, and relative area percentages of the deconvoluted N 1s peaks obtained from XPS analyses of RF-CNO-C, RFM-CNO-C, BX-CNO-C, and CLX-CNO-C samples.

| **Region** | **Species** | **RFM-CNO-C** | | | | **BX-CNO-C** | | | **CLX-CNO-C** | | |
| --- | --- | --- | --- | --- | --- | --- | --- | --- | --- | --- | --- |
|  |  | **Peak (eV)** | **FWHM (eV)** | **% Area** | **Peak (eV)** | | **FWHM (eV)** | **% Area** | **Peak (eV)** | **FWHM (eV)** | **% Area** |
| **N 1s A** | -NH_2_ | 398.56 | 1.50 | 50.3 | 398.22 | | 1.50 | 52.1 | 398.56 | 1.50 | 40.5 |
| **N 1s B** | Protonated amine | 400.75 | 1.48 | 49.7 | 400.55 | | 1.48 | 48.0 | 400.84 | 1.47 | 24.1 |
| **N 1s C** | Imine | - | - | - | - | | - | - | 399.58 | 1.50 | 35.4 |

# Table S5. Specific capacitance, energy, and power values calculated from the GCD studies.

| **Material** | **Current density (A g^-1^)** | ***C_S_* (F g^-1^)** | ***E_density_*  (Wh kg^-1^)** | ***P_density_*  (W kg^-1^)** |
| --- | --- | --- | --- | --- |
| **RF-C** | 6  5  4  3  2 | 23  39  46  50  54 | 1  1  2  2  2 | 1409  1194  946  681  441 |
| **RF-CNO-C** | 6  5  4  3  2 | 31  48  60  72  85 | 1  2  2  2  3 | 1909  1473  1235  804  473 |
| **RFM-C** | 6  5  4  3  2 | 29  36  47  54  69 | 1  1  1  2  2 | 1768  1464  1156  741  470 |
| **RFM-CNO-C** | 6  5  4  3  2 | 108  123  138  149  160 | 4  4  5  5  5 | 1890  1367  996  653  427 |

#

# Table 6. Comparison of the polymer-derived carbon nanostructure-based materials

| **Structure formed** | **Carbon nanostructure** | **Polymeric precursor** | **Surface area [m^2^ g^-1^]** | **specific capacitance** | **electrode**  **system** | **Ref.** |
| --- | --- | --- | --- | --- | --- | --- |
| N-doped mesoporous carbon/graphene composite | graphene | resol, urea | 1348 | 246 F g^−1^ (1.0 A g^−1^) | 3-electrode | ^7^ |
| monolithic mesoporous carbon/graphene aerogels | graphene | resorcinol, formaldehyde | 534 | 120 F g^−1^ (0.5 A g^-1^) | 3-electrode | ^8^ |
| N-doped mesoporous carbon layers anchored on graphene | graphene | resorcinol, formaldehyde | 740 | 314 F g^−1^ (1 A g^−1^) | 3-electrode | ^9^ |
| N-doped carbon sphere and holey graphene hydrogel | graphene | resorcinol, formaldehyde | 338 | 180 F g^−1^ (1.0 A g^−1^) | 3-electrode | ^10^ |
| mesoporous carbon/graphene composite | graphene | phenol, formaldehyde, KOH | 2109 | 329.5 F g^-1^ (0.5 A g^-1^) | 3-electrode | ^11^ |
| mesoporous carbon scaffold on carbon nanotube | carbon nanotubes | phenol, formaldehyde | 614 | 216 F g^-1^ (1 A g^−1^) | 3-electrode | ^12^ |
| mesoporous carbon nanocomposites | carbon nano-onion | *star*-polymer, formaldehyde | 247 | 139 F g^-1^ (0.1 A g^−1^) | 3-electrode | ^13^ |
| mesoporous carbon nanocomposites | carbon nano-onion | *star*-polymer | 74 | 83 F g^-1^ | 3-electrode | ^14^ |
| mesoporous carbon sandwiched graphene nanosheet | graphene | 3-aminophenol, formaldehyde | 989 | 249 F g^−1^ (1.0 A g^−1^) | 2-electrode | ^15^ |
| graphene-containing ordered mesoporous carbon | graphene | resorcinol, hexamethylenetetramine | 1072 | 209 F g^-1^ (0.1 A g^−1^) | 2-electrode | ^16^ |
| mesoporous carbons loading on sulfonated graphene | graphene | phenol, formaldehyde | 1709 | 314 F g^−1^ (1.0 A g^−1^)  261 F g^−1^ (1.0 A g^−1^) | 3-electrode  2-electrode | ^17^ |
| graphene/nitrogen-doped mesoporous carbon nanosheet | graphene | resol, dicyandiamide | 1569 | 377 F g^−1^ (0.2 A g^−1^)  307 F g^−1^ (0.2 A g^−1^) | 3-electrode  2-electrode | ^18^ |
| sulfonated graphene/N-doped mesoporous carbon | graphene | resorcinol, mesitylene, hexamine | 1040 | 304 F g^−1^ (1.0 A g^−1^)  273 F g^-1^ (1.0 A g^-1^) | 3-electrode  2-electrode | ^19^ |
| ordered mesoporous carbons loading on graphene | graphene | phenol, formaldehyde, K_2_CO_3_ | 1309 | 332.5 F g^−1^ (1.0 A g^−1^)  256 F g^−1^ (1.0 A g^−1^) | 3-electrode  2-electrode | ^20^ |
| resin-CNO based carbon nanocomposites | carbon nano-onion | resorcinol, formaldehyde | 723 | 165 F g^−1^  85 F g^−1^ (2.0 A g^-1^) | 3-electrode  2-electrode | This work |
| resin-CNO based carbon nanocomposites | carbon nano-onion | resorcinol, formaldehyde, melamine | 923 | 278 F g^−1^  160 F g^−1^ (2.0 A g^-1^) | 3-electrode  2-electrode | This work |

**
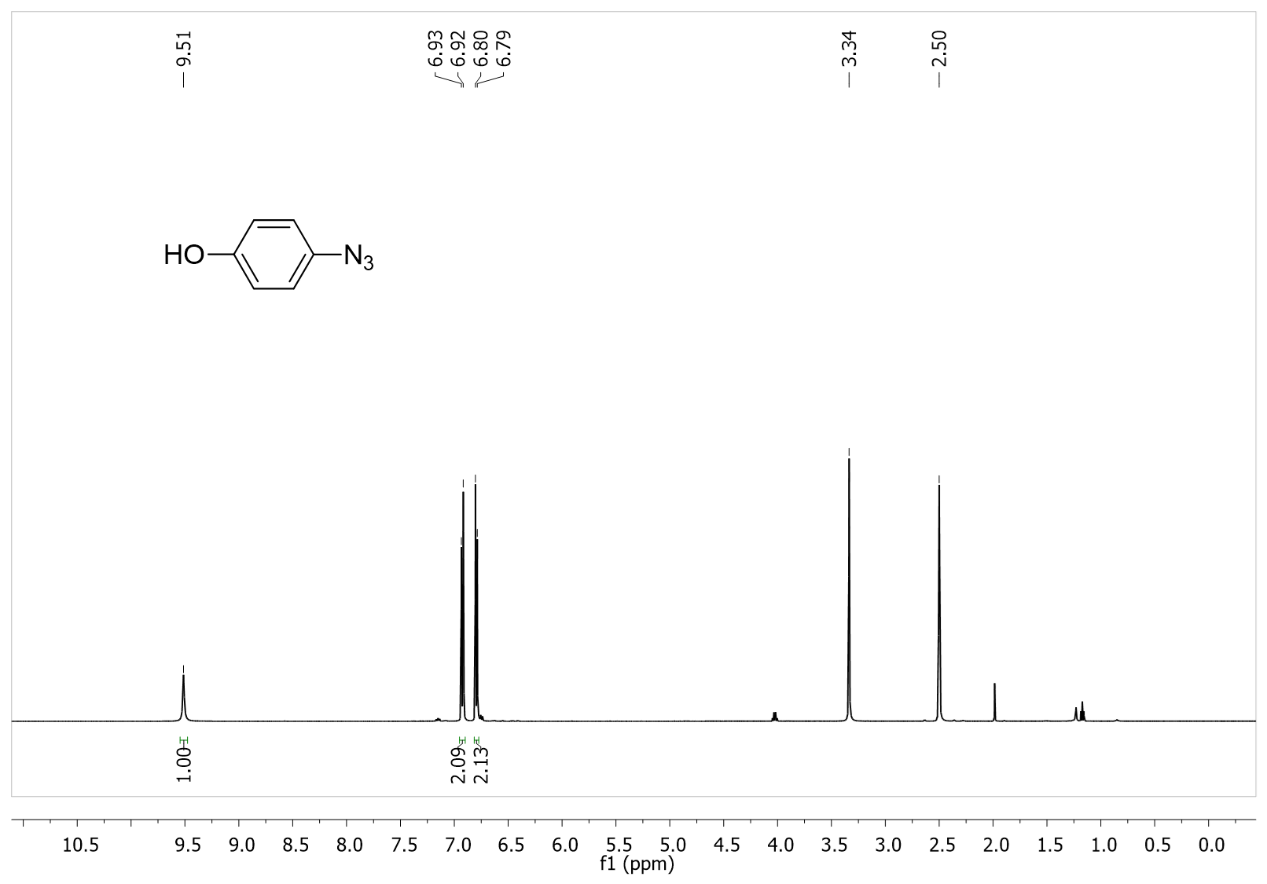
**

# Figure S1. ^1^H NMR spectrum of *p*-azidophenol (500 MHz, CDCl_3_).

**
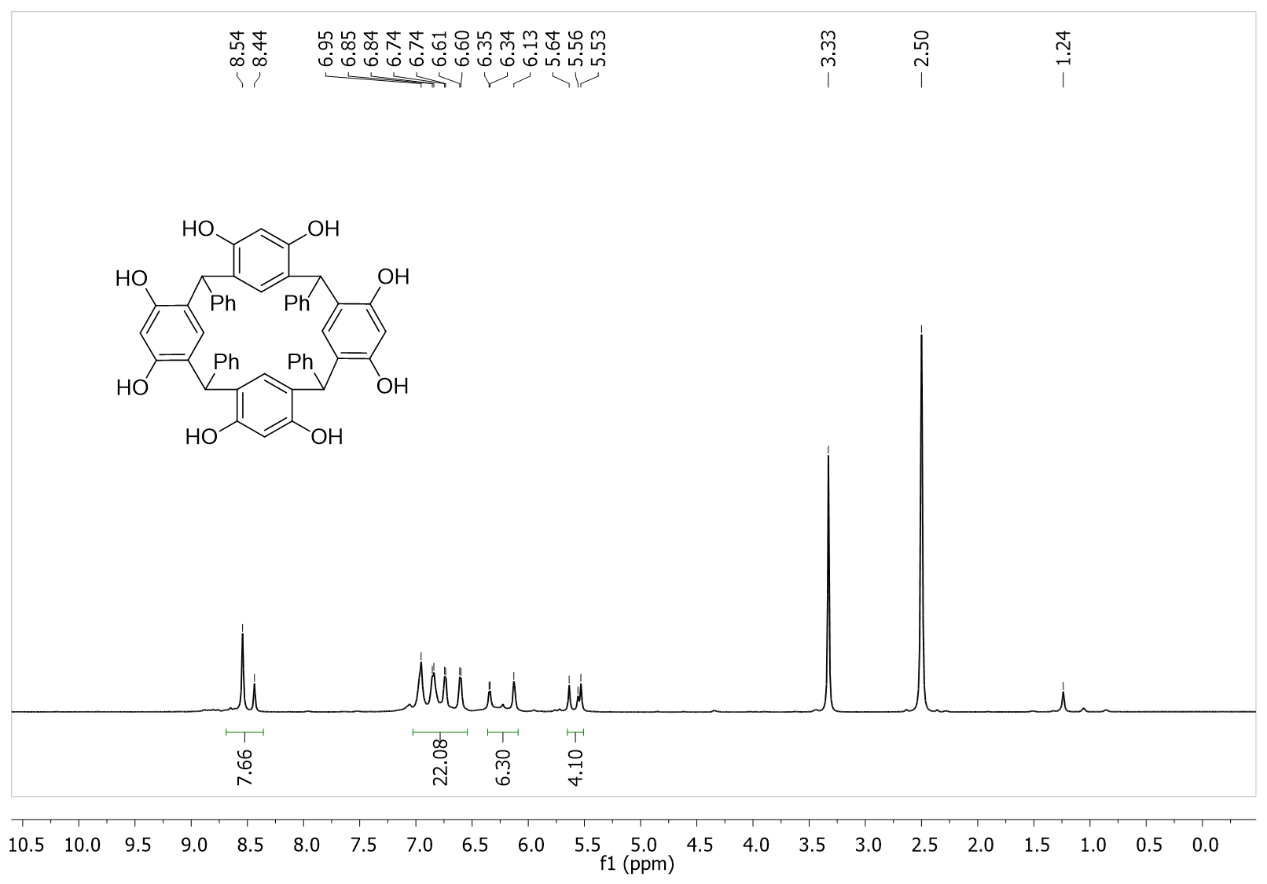
**

# Figure S2. ^1^H NMR spectrum of phenylcalix[4]resorcinarene (500 MHz, DMSO-*d_6_*).


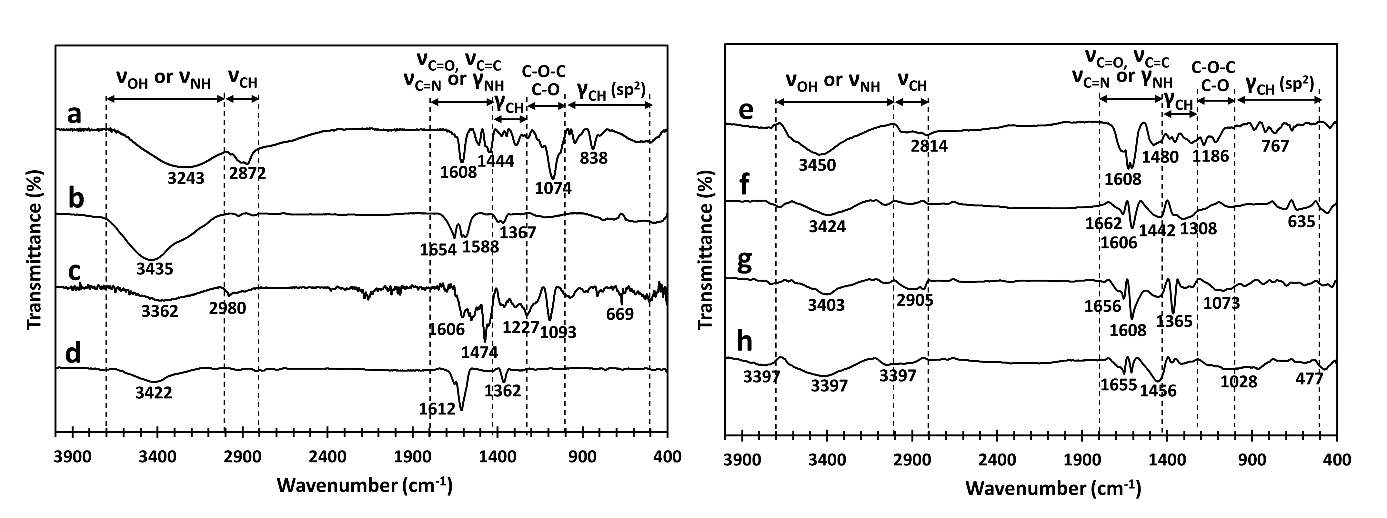


# Figure S3. FTIR spectra of (a) RF, (b) RF-C, (c) RFM, (d) RFM-C, (e) BX, (f) BX-C, (g) CLX, (h) CLX-C.


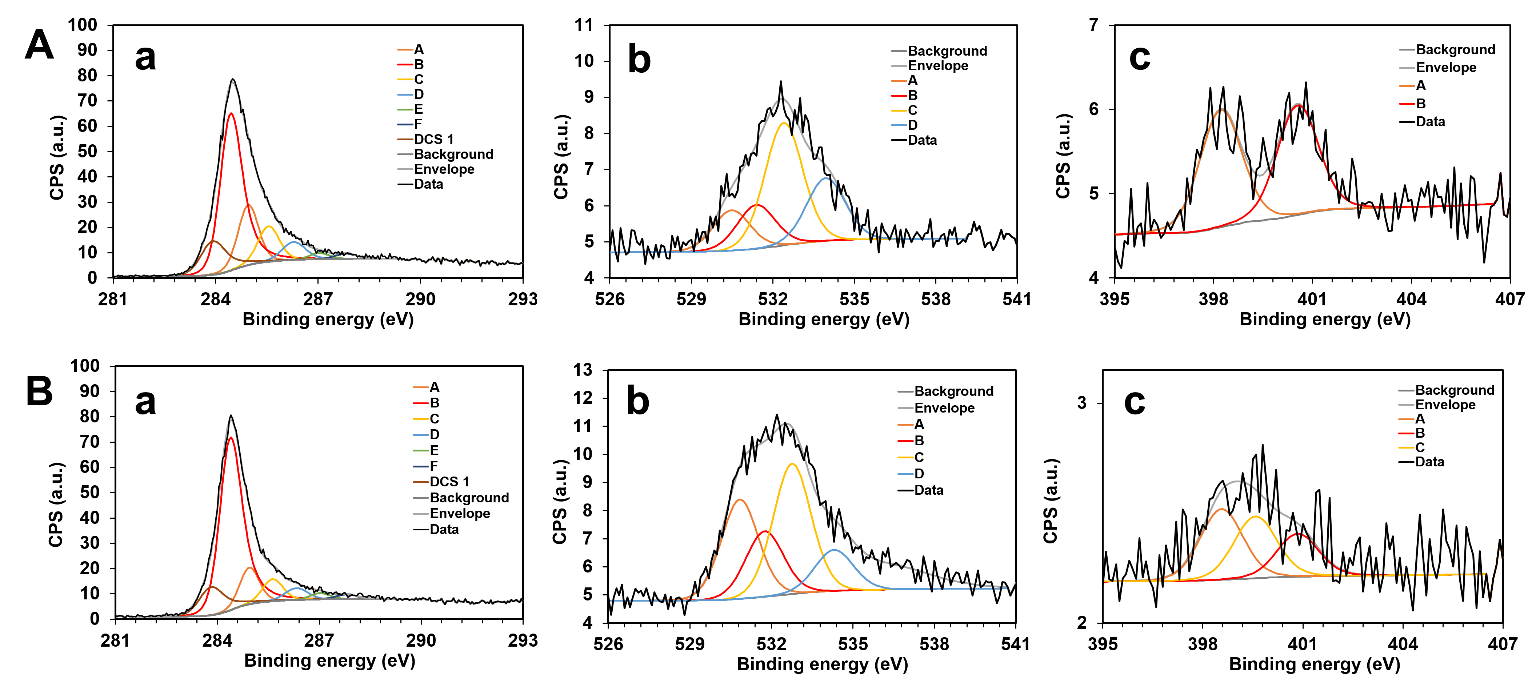


# Figure S4. XPS spectra of the (A) BX-CNO-C and (B) CLX-CNO-C spectral regions (a) C 1s, (b) O1 s, and (c) N 1s.


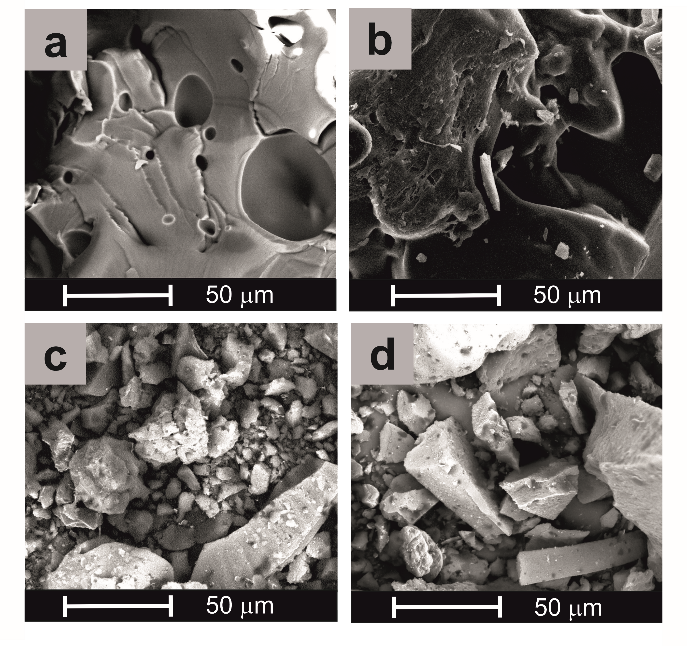


# Figure S5. SEM images of (a) RF, (b) RF-CNO, (c) RFM, (d) RFM-CNO.

# References

1. Courme, C. *et al.* Terminal Alkyne-functionalized Triazine by Sonogashira Coupling: Synthesis of a Potential Cell Signalling Inhibitor via Click Chemistry. *Tetrahedron Lett* **49**, 4542–4545 (2008).

2. Castillo-Aguirre, A. *et al.* Surface Modification of Poly(GMA-co-EDMA-co-MMA) with Resorcarenes. *J Braz Chem Soc* **29**, (2018).

3. Xu, J., Wang, A. & Zhang, T. A Two-step Synthesis of Ordered Mesoporous Resorcinol–formaldehyde Polymer and Carbon. *Carbon* **50**, 1807–1816 (2012).

4. Lu, C. *et al.* The Effects of Melamine on the Formation of Carbon Xerogel Derived From Resorcinol and Formaldehyde and Its Performance for Supercapacitor. *J Colloid Interface Sci* **524**, 209–218 (2018).

5. Katanyoota, P., Chaisuwan, T., Wongchaisuwat, A. & Wongkasemjit, S. Novel Polybenzoxazine-based Carbon Aerogel Electrode for Supercapacitors. *Mater Sci Eng B* **167**, 36–42 (2010).

6. Abadast, F., Mouradzadegun, A. & Ganjali, M. R. Rational Design, Fabrication and Characterization of a Thiol-rich 3D-porous Hypercrosslink Polymer as a New Engineered Hg^2+^ Sorbent: Enhanced Selectivity and Uptake. *New J Chem* **41**, 5458–5466 (2017).

7. Sui, L. *et al.* N-doped ordered mesoporous carbon/graphene composites with supercapacitor performances fabricated by evaporation induced self-assembly. *Int. J. Hydrog. Energy* **42**, 29820–29829 (2017).

8. Chandrasekaran, N., Premkumar, V., Senthil Kumar, S. M. & Ram, R. Single-step rapid synthesis of monolithic mesoporous carbon/graphene aerogels with improved double layer capacitance. *New J. Chem.* **42**, 7371–7376 (2018).

9. Shen, Z., Du, J., Mo, Y. & Chen, A. Nanocomposites of reduced graphene oxide modified with mesoporous carbon layers anchored by hollow carbon spheres for energy storage. *Carbon* **173**, 22–30 (2021).

10. Mi, M. *et al.* Hierarchical composite of N-doped carbon sphere and holey graphene hydrogel for high-performance capacitive deionization. *Desalination* **464**, 18–24 (2019).

11. Song, Y., Li, Z., Guo, K. & Shao, T. Hierarchically ordered mesoporous carbon/graphene composites as supercapacitor electrode materials. *Nanoscale* **8**, 15671–15680 (2016).

12. Wang, Y. *et al.* Nitrogen-doped porous carbon monoliths from polyacrylonitrile (PAN) and carbon nanotubes as electrodes for supercapacitors. *Sci. Rep.* **7**, 40259 (2017).

13. Siemiaszko, G. *et al.* Polymeric Network Hierarchically Organized on Carbon Nano-onions: Block Polymerization as a Tool for the Controlled Formation of Specific Pore Diameters. *ACS Appl. Polym. Mater.* **4**, 2442–2458 (2022).

14. Siemiaszko, G., Hryniewicka, A., Breczko, J., Brzezinski, K. & Plonska-Brzezinska, M. E. Carbon nano-onion induced organization of polyacrylonitrile-derived block star polymers to obtain mesoporous carbon materials. *Chem. Commun.* **58**, 6829–6832 (2022).

15. Liu, Y. *et al.* Single-Layered Mesoporous Carbon Sandwiched Graphene Nanosheets for High Performance Ionic Liquid Supercapacitors. *J. Phys. Chem. C* **121**, 23947–23954 (2017).

16. Qiu, J., Wang, Y., Wu, F., Liu, W. & Zhang, S. Graphene-containing ordered mesoporous carbons synthesized by one-pot aqueous route and its electrochemical performance. *Polym. Compos.* **38**, 1438–1446 (2017).

17. Lu, S., Guo, K., Xie, Y. & Ning, J. Ordered Mesoporous Carbons Loading on Sulfonated Graphene by Multi-Components Co-Assembly for Supercapacitor Applications. *Energy Technol.* **6**, 1975–1985 (2018).

18. Song, Y. *et al.* In-situ synthesis of graphene/nitrogen-doped ordered mesoporous carbon nanosheet for supercapacitor application. *Carbon* **96**, 955–964 (2016).

19. Chen, P., Yang, C., He, Z. & Guo, K. One-pot facile route to fabricate the precursor of sulfonated graphene/N-doped mesoporous carbons composites for supercapacitors. *J. Mater. Sci.* **54**, 4180–4191 (2019).

20. Chen, P. *et al.* Ordered Mesoporous Carbons Loading on Graphene after Different Molten Salt Activations for Supercapacitor Applications. *Energy Technol.* **6**, 2273–2281 (2018).
