# Supplementary material for: Composites containing resins and carbon nano-onions as efficient porous carbon materials for supercapacitors
Source: Sci Rep. 2023 Apr 24;13:6606. doi: 10.1038/s41598-023-33874-w (PMC10126139; doi:10.1038/s41598-023-33874-w)
Supplement: Supplementary file 2 — Supplementary Information 2. [file 41598_2023_33874_MOESM2_ESM.docx]

**Supporting Information**

The following files are available free of charge. Procedures of synthesis of *p*-azidophenol, obtaining resins: **RF**, **RFM**, **BX** and **CLX**, and their pyrolysis; ^1^H NMR spectra of *p*-azidophenol and CLX; XPS spectra of C 1s, N 1s, O 1s of **BX-CNO-C** and **CLX-CNO-C**, surface elemental composition, chemical state, positions, and relative area percentages of the deconvoluted C 1s, O 1s, and N 1s peaks obtained from XPS analyses of all samples; specific capacitance, energy, and power values calculated from GCD studies for **RF-C**, **RF-CNO-C**, **RFM-C**, and **RFM-CNO-C**, as well as SEM images of **RF**, **RF-CNO**, **RFM**, and **RFM-CNO** (PDF).
